# Validation of seismic hazard curves using a calibrated 14 ka lacustrine record in the Eastern Alps, Austria

**DOI:** 10.1038/s41598-022-24487-w

**Published:** 2022-11-19

**Authors:** Christoph Daxer, Jyh-Jaan Steven Huang, Stefan Weginger, Michael Hilbe, Michael Strasser, Jasper Moernaut

**Affiliations:** 1 Institute of Geology, University of Innsbruck, Innrain 52f, 6020 Innsbruck, Austria; 2 Institute of Oceanography, National Taiwan University, Taipei, Taiwan; 3 ZAMG–Zentralanstalt für Meteorologie und Geodynamik, Vienna, Austria; 4 Institute of Geological Sciences and Oeschger Centre of Climate Change Research, University of Bern, Bern, Switzerland

**Keywords:** Natural hazards, Solid Earth sciences

## Abstract

Seismic hazard maps are crucial for earthquake mitigation and mostly rely on probabilistic seismic hazard analysis (PSHA). However, the practise and value of PSHA are under debate because objective testing procedures for seismic hazard maps are scarce. We present a lacustrine turbidite record revealing 44 earthquakes over the last ~ 14 ka and use it to test seismic hazard curves in southern Austria. We derive local seismic intensities for paleo-earthquakes by applying scaling relationships between the sedimentary imprint and seismic intensity of well-documented historical earthquakes. The last ~ 2.8 ka of the record agree with a Poissonian recurrence behaviour and therefore a constant hazard rate, which is the modelling choice for standard PSHA. The lacustrine data are consistent with the intensity-frequency relationship of the local seismic hazard curve, confirming the current PSHA approach for this part of Austria. On longer timescales, distinct phases of enhanced regional seismicity occurred, indicating a potential increase of seismic hazard after large earthquakes—a factor hitherto disregarded in the PSHA of the Eastern Alps. Our new method forms an independent procedure to test hazard maps in any setting where suitable lake systems are available.

## Introduction

Seismic hazard maps aim at providing communities with information about earthquake hazards and form the basis to develop appropriate mitigation strategies. Current best-practice seismic hazard maps mainly rely on probabilistic seismic hazard analysis (PSHA). PSHA combines models for the size and location of potential earthquakes and ground-motion models to forecast the shaking strength expected with a certain probability within a given period. The underlying assumption of standard probabilistic hazard modelling is that earthquake occurrence follows a time-independent Poissonian process, which implies a constant hazard rate over time^1^. On individual fault segments, however, strong earthquakes often occur in a more regular (quasi-periodic) pattern or in clusters with short interevent times separated by long quiescence periods^2^. PSHA has also been subject to criticism because of the occurrence of large, damaging earthquakes in regions of relatively low hazard according to seismic hazard maps^3,4^. The 2011 Tohoku earthquake (M 9.0), for instance, severely affected areas where the Japanese hazard map of 2010 predicted a probability of less than 0.1% of shaking with a minimum intensity “6-lower” on the Japan Meteorological Agency seismic intensity scale in the following 30 years^5^. Mitigation methods therefore were not prepared for an event of this magnitude and the subsequent tsunami, resulting in ~ 19,000 dead or missing persons and a direct damage of ~ $210 billion^6^. However, due to the probabilistic nature of the seismic hazard maps, individual earthquakes with shaking stronger than mapped do not invalidate a map^7,8^, but give reason to re-evaluate the PSHA input parameters and methodology, and to develop approaches to thoroughly test the resulting maps.

Assessing the performance of seismic hazard maps mainly resorts to the validation of the individual hazard model components and its input data (see Gerstenberger et al.^9^ and references therein). On-fault geologic and geomorphic studies have contributed greatly to seismic hazard analysis by providing data about the location, geometry and kinematics of active faults, information about their slip rate as well as the timing and magnitude of previous ruptures^10^. However, in regions where surface ruptures are absent or hardly accessible (e.g. subduction megathrust faults) or areas where strain is accommodated by complex fault networks (e.g. intraplate regions), the common methods to obtain this geological data (paleoseismic trenching, offset markers) are not applicable. Also, on-fault paleoseismology only informs fault source models and cannot validate the overall adequacy of a seismic hazard map in terms of seismic shaking.

A direct testing of seismic hazard curves has been attempted by using written historical data^11^. However, these (1) often fail to capture the long (> 500–1000 years) and variable recurrence times of the largest earthquakes in a region, especially in slowly deforming intraplate regions, and (2) often are used as model input data, and therefore cannot be used for model validation^9,12^. Short individual (paleo-) earthquakes chronologies can produce biased recurrence statistics^13,14^ and make it hard to assess hazard map performance^7^. A common approach is therefore to integrate observations from several sites and substitute time for space^15–17^. For this method to be valid, however, spatial dependence of the observations must be ruled out or accounted for^18^.

Lake sediments can archive seismic shaking with intensities of ~ V or higher^19–21^ as mass-transport deposits (MTDs), turbidites or in-situ deformations. Due to continuous sedimentation, they often provide exceptional age control and sensitively record several thousand years of earthquake shaking occurrence^22–25^. In case the sedimentary imprints are properly calibrated with shaking data from historical and/or recent earthquakes^20,26^, such lacustrine off-fault paleoseismic records can present a long-term archive of seismic ground motion and thus enable direct testing of hazard curves that underly seismic hazard maps.

Here, we use a calibrated lacustrine record spanning 14,000 years to independently test a local seismic hazard curve in Southern Austria. To do so, we verify whether the lake paleoseismic record may reflect a Poissonian process, as this is the underlying assumption of PSHA in Austria. We then compare the intensity-frequency relationships obtained from the lake record with the local seismic hazard curve and discuss how this new method can be used for independent validation and improvement of probabilistic seismic hazard analysis.

## Seismotectonic framework and lake setting

**Figure 1:**
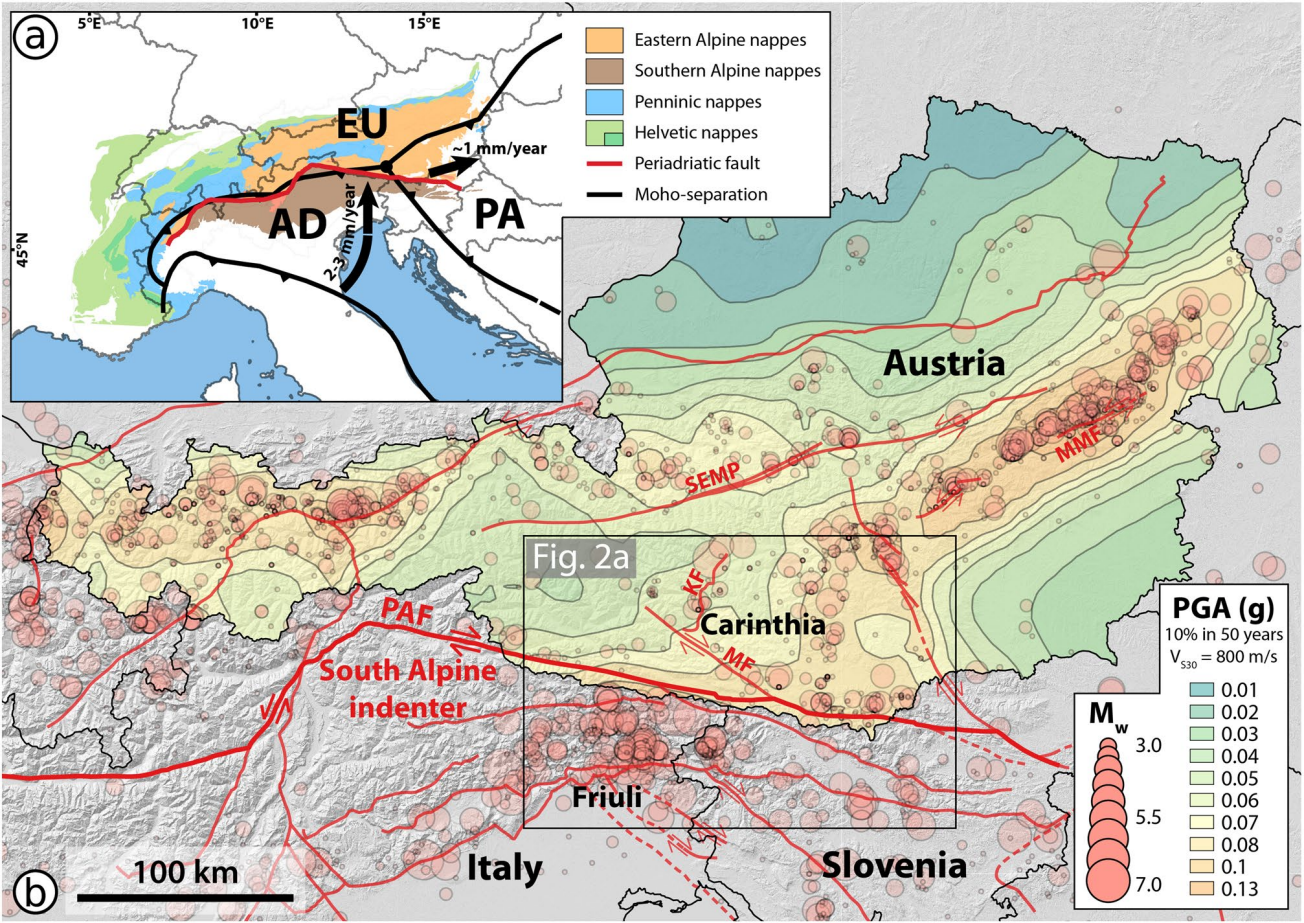
Seismotectonic setting of the study area. (**a**) Tectonic map (simplified after Schmid et al.^71^) and crustal structure of the European Alps (modified after Brückl et al.^27^ and Faccenna et al.^72^). Arrows indicate the relative motion of the Adriatic plate (AD) and the Pannonian fragment (PA) relative to the European plate (EU). (**b**) Current seismic hazard map of Austria^29^. KF, Katschberg normal fault; MF, Mölltal fault; MMF, Mur-Mürz fault; PAF, Periadriatic fault; SEMP, Salzach-Ennstal-Mariazell-Puchberg fault. Earthquake locations and magnitudes are plotted according to the Austrian earthquake catalogue^34^. The maps were generated using QGIS software (version 3.20.2; http://www.qgis.org).

**Figure 2:**
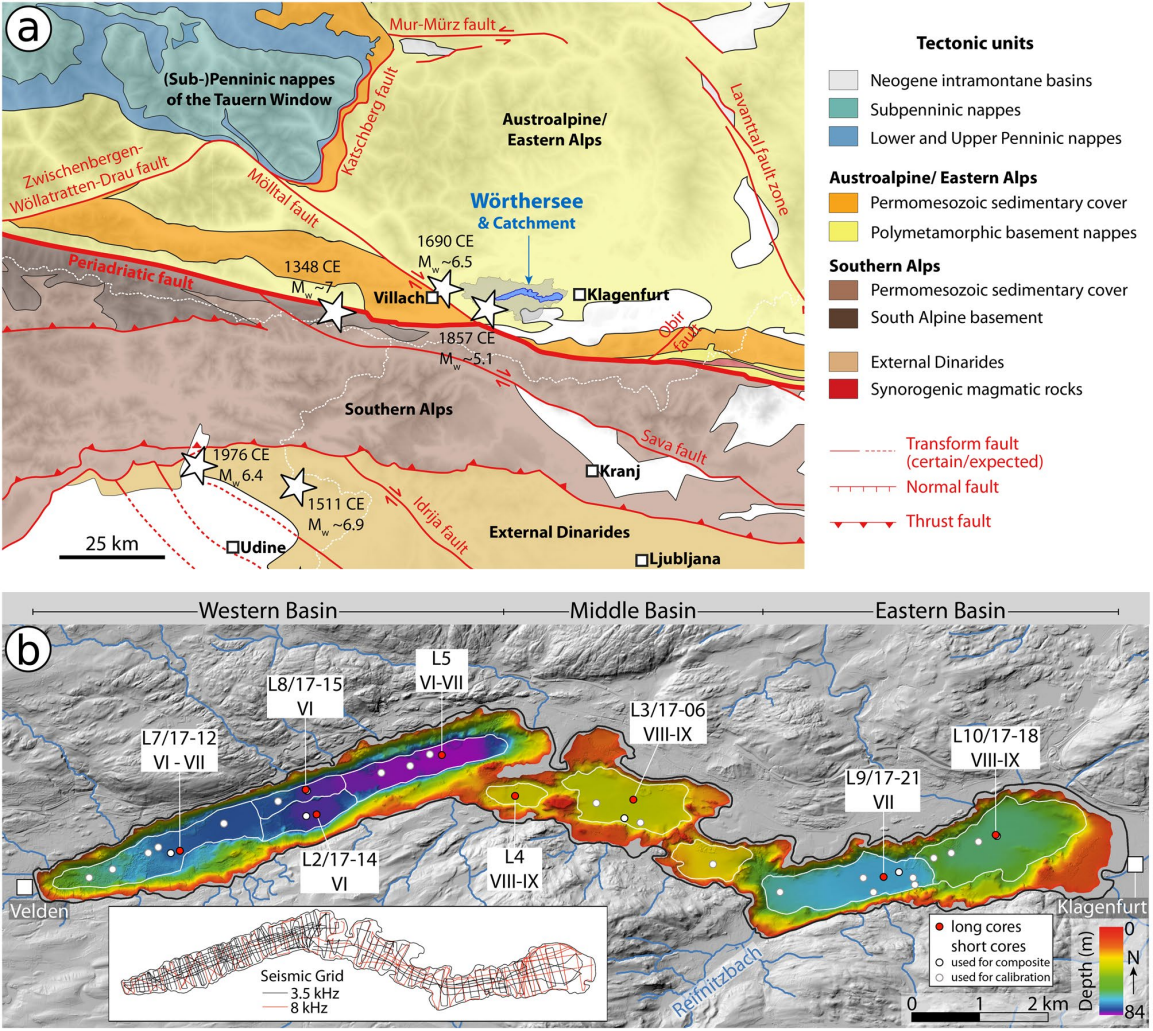
Overview of the study area. (**a**) Seismotectonic setting (simplified from Reinecker and Lenhardt^73^, Reiter et al.^74^, and Schmid et al.^71^)*.* Earthquake epicentres and magnitudes were compiled from SHEEC 1000-1899^32^, SHEEC 1900-2006^31^ and the Austrian earthquake catalogue^34^. (**b**) Bathymetric map of Wörthersee. Coring locations are indicated as red (long Kullenberg cores) and white (short percussion cores) dots. Each coring location has a specific intensity threshold for recording earthquakes, given in roman numerals^20^. Different depositional areas are outlined in white (simplified after^20^). In the lower left, the acquired reflection seismic grid using two different seismic sources is shown. The maps were generated using QGIS software (version 3.20.2; http://www.qgis.org).

The studied lake Wörthersee (See = German for lake) is located in the Austrian federal state of Carinthia, close to the border of Slovenia and Italy (Fig. 1). The crustal structure of this area presents three lithological domains: the European plate, the Adriatic microplate and the Pannonian fragment^27^ (Fig. 1a). The counterclockwise rotation of the Adriatic microplate that is ongoing since ~ 35 Ma causes a present-day N-S convergence of ~ 2–3 mm/year in the Eastern Alps. This convergence is accommodated by (1) the indentation of the relatively rigid Adriatic microplate (so-called South Alpine indenter) into the European plate, separated at the surface by the Periadriatic fault (PAF); (2) uplift in the Eastern Alps and (3) eastward extrusion of the Pannonian fragment^27,28^ (Fig. 1a, b). This geodynamic setting is reflected by back-thrusting in the southern Alps and strike-slip faults in the Eastern Alps and the Dinarides (Figs. 1b, 2a).

Carinthia is among the regions with the highest seismic hazard in Austria (Fig. 1b) due to its position close to the highly seismically active areas of Friuli and western Slovenia, seismicity along the Katschberg-, Mölltal- and Periadriatic fault as well as diffuse seismicity^29^. Historical and instrumental seismicity data attest that the area of Wörthersee has experienced at least five earthquakes of local intensities (I_L_) > V on the European Macroseismic scale (EMS-98^30^) over the last ~ 800 years^31,32^: in 1976 (M_w_ 6.4; I_L_ V–V½), 1857 (M_w_ ~ 5.1; I_L_ ~ V½), 1690 (M_w_ ~ 6.5; I_L_ VII½–VIII½), 1511 (M_w_ ~ 6.9; I_L_ VI¾–VII¾) and 1348 (M_w_ ~ 7; I_L_ VIII–IX). The latter presumably constitutes the largest documented historical earthquake in the Alps.

Wörthersee (area = 19 km^2^, depth = 84 m) is a glacigenic lake fed by rather small inflows (mean discharge of the main inflow Reifnitzbach is 0.63 m^3^/s), draining a total catchment area of 162 km^2^. The lake is usually meromictic, i.e. mixing of the water column is limited to a certain depth, but oxygen-rich bottom water has been observed in recent years^33^. Wörthersee is subdivided into three main parts (western, middle, and eastern), which are in turn divided into physically separated depositional areas (DAs) by bedrock ridges or glacial morphologies (Fig. 2b).

## Methods

### Seismic hazard analysis in Austria

#### Earthquake catalogue

The underlying data source of seismic hazard analysis in Austria is the Austrian Earthquake Catalogue (AEC^34^), which covers the last ~ 1000 years. Outside of Austria, the AEC was harmonized with the catalogue of the International Seismological Centre (ISC). Due to the limited catalogue completeness in Carinthia (determined using the slope methods of Hakimhashemi and Grünthal^35^ and Stepp^36^), only the last ~ 200 years were used for the calculation of the magnitude-frequency-relationship in the Wörthersee source zone, thus excluding the strong historical earthquakes of 1690, 1511, and 1348CE. The minimum magnitude considered in PSHA was 3, and the catalogue was declustered following Gardner and Knopoff^37^, leading to a time window of ~ 1.4 years (for M = 6) within which an event is identified as part of an earthquake cluster.

#### Seismic hazard map

The current national seismic hazard map of Austria^29^ (Fig. 1b) is based on standard probabilistic seismic hazard analysis. The PSHA relies on (1) recently developed relationships between local magnitude, moment magnitude (M_w_) and epicentral intensity and (2) a set of regional and global ground motion prediction equations (GMPEs), selected by statistical parameters from local seismic recordings. The input model for the hazard analysis is composed of two area source models and a smoothed seismicity model. In addition, a large-scale area source model is used to compensate for insufficient local data. For each source zone, the mean earthquake parameters (e.g. depth-distribution and source-mechanism) are calculated. The seismicity rate (magnitude-frequency distributions) of each source zone is determined using common methods^38^ and Bayesian penalized maximum likelihood^39^. Estimations of the maximum magnitude (M_max_) are based on the Electric Power Research Institute (EPRI) approach^40^, which was developed for areas with moderate seismicity. All models, magnitude frequency distributions, M_max_ and GMPEs are combined in a logic tree and PSHA was performed with the software OpenQuake^41^. The output are hazard curves indicating the probability of exceedance of the maximum horizontal acceleration^29^.

### Lacustrine paleoseismology

#### Hydro-acoustic methods

High-resolution bathymetric data (1 m) was acquired on Wörthersee using a SeaBat T50-P multibeam echosounder in combination with an AsteRx-U MARINE GNSS Heading System in 2017. Reflection seismic data were acquired using a single-channel 3.5 kHz Kongsberg Geopulse pinger (theoretical vertical resolution ~ 10 cm) and an Innomar SES-2000 light subbottom profiler (100 kHz primary frequency, 8 kHz secondary frequency; theoretical vertical resolution ~ 5 cm; see Fig. 2b for traces of seismic profiles). Seismic interpretation was done in IHS Markit Kingdom Suite version 2020, applying a bandpass filter (2–6 kHz) to the 3.5 kHz seismic data. Mass-transport deposits (MTDs) and their equivalent seismic-stratigraphic horizons were mapped to recognise synchronous—and therefore likely earthquake-triggered^25^—subaqueous slope failures. Thickness grids of MTDs (Supplementary Figs. 1–4) were calculated using the software Surfer 10 (Golden Software; simple kriging interpolation) by assuming an acoustic velocity of 1500/ms.

#### Sediment core analyses

In 2017 and 2018, short (~ 1.5 m) sediment cores were acquired in Wörthersee using a gravity corer with a manual percussion system. In 2018, long (up to ~ 11 m) sediment cores were retrieved with a modified Kullenberg gravity piston coring system^42^ and cut into 1.5 m sections. X-ray computed tomography (CT) scanning was carried out at the Medical University of Innsbruck, using a Siemens SOMATOM Definition AS with a voxel size of 0.2 × 0.2 × 0.3 mm. γ-density (at 0.5 cm resolution) and magnetic susceptibility data (at 0.2 cm resolution) was acquired at the Austrian Core Facility using a GEOTEK Multi-Sensor Core Logger (MSCL) and a Bartington MS2E point sensor. To assess elemental variability, the cores were scanned at a resolution of 0.2 or 1 mm with an ITRAX X-ray fluorescence scanner using a Mo tube (30 kV, 45 mA, 5 s exposure time). A centered-log ratio transformation was applied to the elemental intensities to avoid possible matrix and asymmetry effects^43^. Based on distinct patterns in the XRF-signal, a detailed core-to-core correlation was achieved.

#### Chronology

To confirm the core-to-core correlation and to date turbidites, AMS^14^C dating was carried out on terrestrial macro-remains from long cores WOER18-L5 and WOER18-L9 and short cores WOER17-02 and WOER17-21 (Supplementary Table 1). All ages were calibrated using IntCAL20^44^ and are reported as calibrated years before present (cal BP). Continuous age-depth models were produced using the Bayesian software BACON v. 2.5.7.^45^ (Supplementary Fig. 5), excluding all event layers > 1 cm. All dates were projected onto core WOER18-L5, resulting in a combined age-depth-model (Supplementary Fig. 5c). Turbidite ages were obtained from this model using a total of 3586 age-model iterations (Supplementary Table 2).

#### Earthquake trigger confidence levels and temporal resolution of turbidites

Turbidites were identified based on their homogeneous or fining-upward macroscopic appearance, high counts in Ca or Ti as well as their colour, which contrasts to the finely laminated hemipelagic background sediment^20^ (Supplementary Figs. 6–15). To infer an earthquake trigger of the turbidites, we rely on the “synchronicity criterion”^21,46,47^: simultaneously deposited turbidites in physically separated basins (= depositional areas) suggest a regional trigger, i.e. an earthquake. This approach is validated in a previous study by the presence of coeval turbidites related to the historical earthquakes of 1976, 1857, 1690, 1511, and 1348 CE^20^. Following this reasoning, we assign an earthquake trigger confidence level to each set of time-correlative turbidites (subsequently called “events”; Supplementary Table 3): The event deposits corresponding to historically documented earthquakes are assigned a very high confidence level; events recorded both in the western and eastern basin are assigned a high confidence level; events recorded in at least two long cores are assigned an intermediate confidence level; and events that are only present in a single long core are assigned a low confidence level. For some recent events, an anthropogenic trigger mechanism was inferred as these link to the construction of coastal infrastructure^48^. These human-induced deposits are discarded as earthquake-triggered, as are events that only show turbidites smaller than 2 mm (very low confidence level).

The deposition of the fine-grained, silty to clayey top of a turbidite takes weeks to months^49^. Two earthquakes with sufficient local seismic intensities that follow each other in close succession (< 1 year) would therefore lead to an amalgamated turbidite^50^, which we would interpret as a single earthquake. Therefore, the lake paleoseismic record of Wörthersee only contains mainshocks.

#### Calculation of local seismic intensities

**Figure 3:**
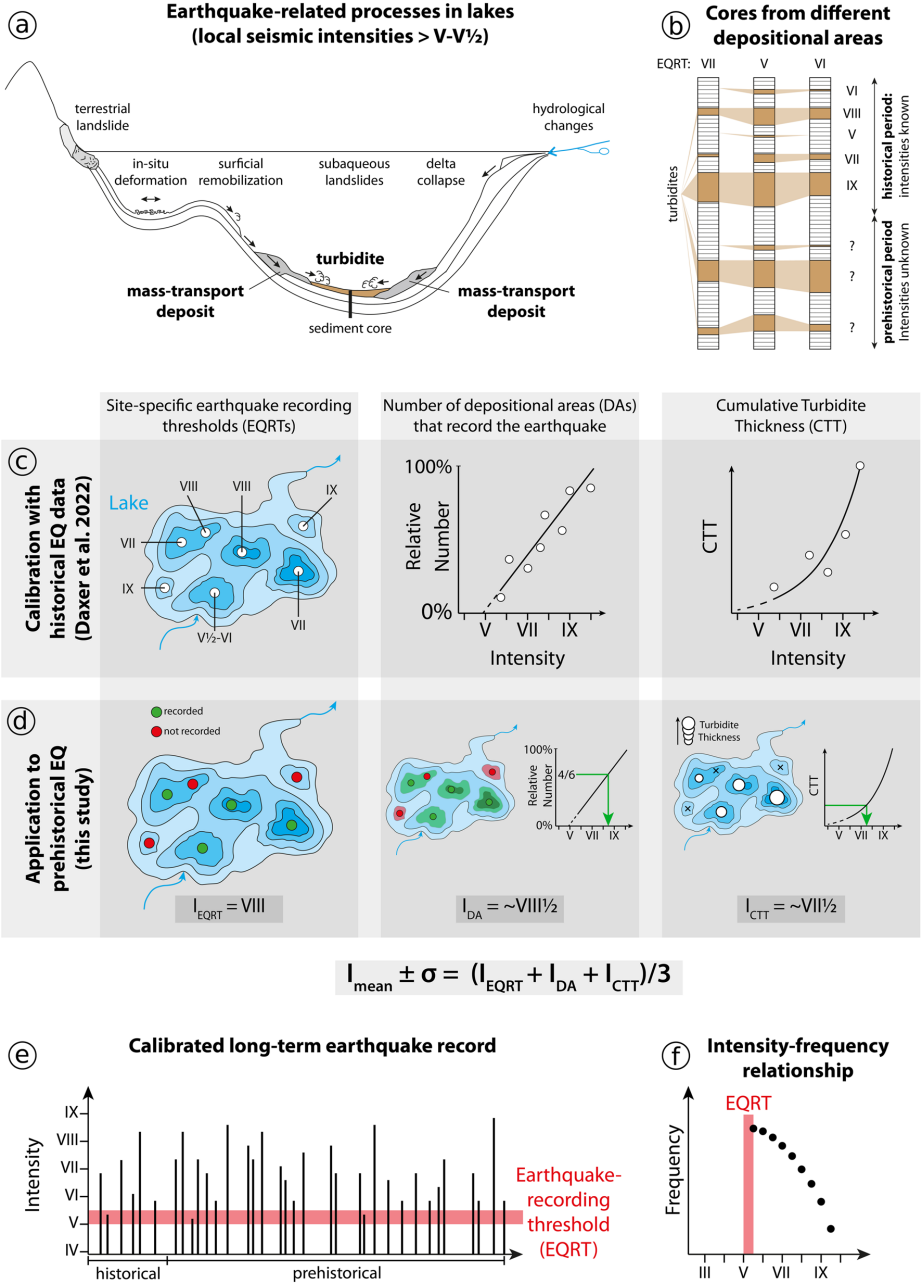
Methodological concept of this study. (**a**) Potential earthquake-induced imprints in lakes (modified after^75^). In Wörthersee, the general earthquake-recording threshold (EQRT, i.e. the seismic intensity above which earthquake-related mass-transport deposits and turbidites are to be expected) is V–V½^20^. (**b**) Cores from different depositional areas (having different, site-specific EQRTs) are retrieved and turbidites identified. (**c**) In addition to site-specific EQRTs (upper left panel), Daxer et al.^20^ derived relationships between seismic intensity at the lake site and (i) the relative number of depositional areas (DAs) that record an earthquake (upper mid panel) and (ii) the cumulative turbidite thickness (CCT; upper right panel). (**d**) In this study, a combination of these intensity indicators is used to derive a “mean intensity” of prehistorical earthquakes recorded in the long sediment cores of Wörthersee. Furthermore, the mean $\pm$ the standard deviation of the calculation is used as an upper (I_max_) and lower intensity limit (I_min_) for each recorded earthquake. (**e**) Thus, a calibrated long-term earthquake record is constructed. Earthquakes with local intensities close to the general EQRT of V–V½ are likely underrepresented. (**f**) The long-term earthquake record is then used to derive the intensity-frequency relationships needed to evaluate the local hazard curve calculated by PSHA.

Based on the historically or instrumentally documented earthquakes of 1976, 1857, 1690, 1511, and 1348 CE that are archived as turbidites and MTDs in the sedimentary record of Wörthersee, Daxer et al.^20^ established site-specific earthquake-recording thresholds (EQRTs) and scaling relationships between the sedimentary imprint and seismic intensity. To derive the local seismic intensity of an earthquake archived as a turbidite in the pre-historic sedimentary record of Wörthersee, we calculate the mean and standard deviation of the three intensity measures I_EQRT_, I_DA_ and I_CTT_, where (1) I_EQRT_ is the intensity derived from the maximum EQRT of coring sites with positive evidence, (2) I_DA_ is the intensity derived from the scaled relative number of depositional areas that show positive evidence, and (3) I_CTT_ is derived from the scaled cumulative turbidite thickness (Fig. 3).

### Recurrence statistics

To account for the influence of age uncertainty on the recurrence data, we used all 3586 age-depth-model iterations and calculated the mean interevent times, the memory (M) and the burstiness (B) coefficient^51^. M ranges from − 1 to 1 and is positive when a long (short) interevent time is tendentially followed by a long (short) interevent time and negative when a long (short) interevent time is followed by a short (long) interevent time. B ranges from − 1 to 1 and provides insights in how regular earthquakes took place, with B = − 1 representing a perfectly periodic recurrence. A Poissonian (“time-independent” or “aperiodic”) process relates to a constant hazard rate and is characterized by an exponential distribution of interevent times for which B = 0. B values larger than 0 indicate a “bursty” recurrence pattern, meaning that the standard deviation of interevent times is larger than their mean. We follow the classification of Kempf and Moernaut^13^ and use the term “bursty” for B > 0.05, “aperiodic” for − 0.05 < B <  + 0.05, “weakly periodic” for − 0.33 < B < − 0.05 and “strongly periodic” for B < − 0.33.

We evaluated whether the recurrence intervals are exponentially distributed—a requisite for a Poissonian process—using two statistical tests: (1) the Lilliefors-corrected Kolmogorov–Smirnov (KS) test (R-package “KScorrect”^52^) and (2) the two-sided Cox and Oakes test for exponentiality (R-package “exptest”^53^). The exponential distribution is rejected (at the 95% confidence level) if the p-value of the test is lower than 0.05. We also tested the goodness-of-fit of several other probability density functions often used in paleoseismic research^54^: gamma, lognormal and Weibull. A lognormal distribution is typical for a bursty recurrence behaviour with a high hazard rate after an event and a monotonic decrease with time. The same is true for gamma and Weibull distributions with α < 1. If α > 1, the gamma and Weibull distributions indicate a seismic cycle with a certain degree of periodicity, exhibiting a near-zero hazard after a large event and increasing with time.

## Results

### Paleoseismic record

**Figure 4:**
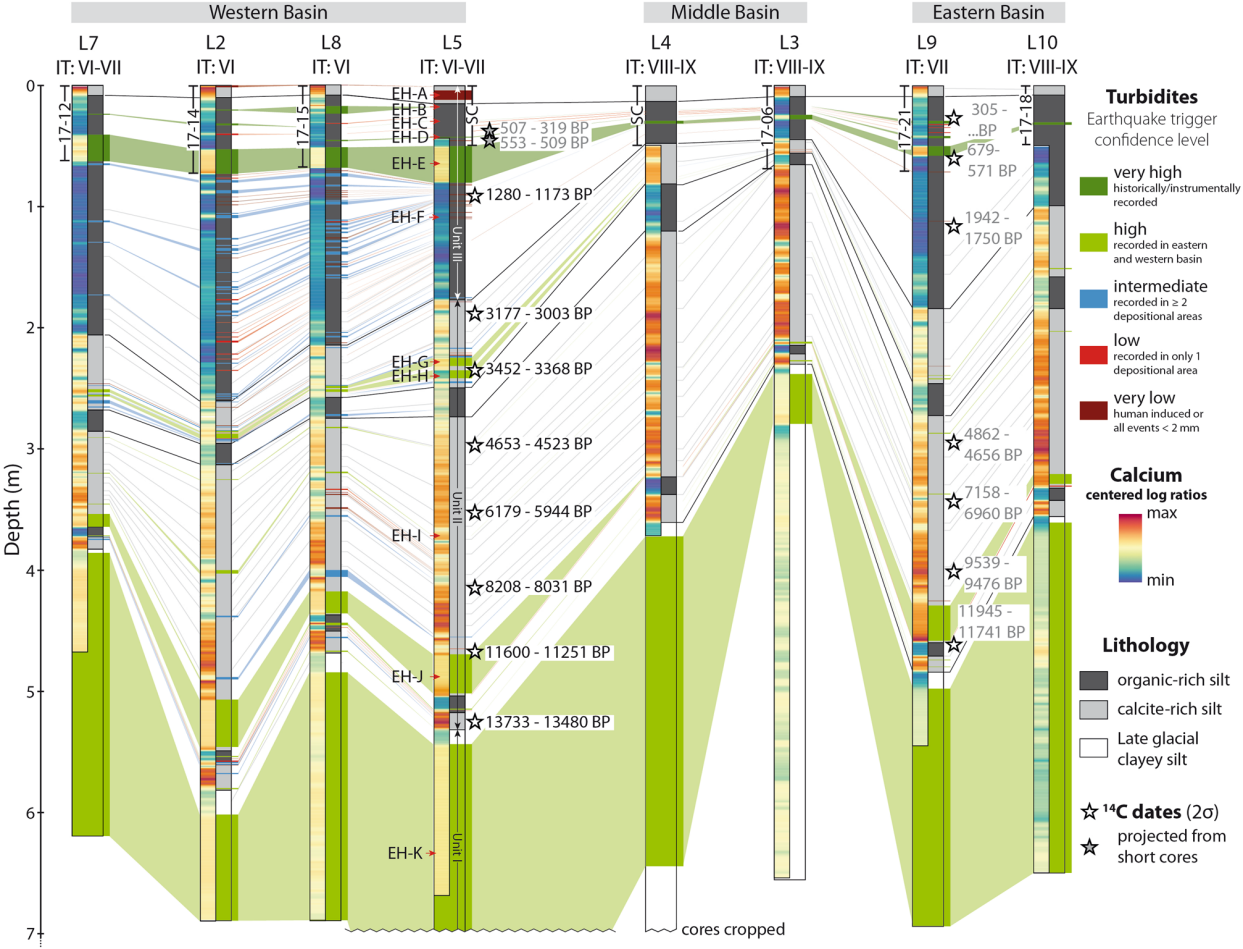
Correlation and general lithology of sediment cores in Wörthersee. The uppermost part of the composite sediment cores consists of short sediment cores (indicated by the core names or “SC” when the Kullenberg trigger core was used). Depending on the distribution of turbidites, we assign an earthquake trigger confidence level to each turbidite-interval. The background-sedimentation can be divided into three main lithologies: (i) partly laminated dark organic-rich silt with relatively low amount of calcite; (ii) laminated calcite-rich silt and iii) grey, laminated late glacial clayey silt^48^. As indicated by the distribution of calcium in the sediment (blue to red palette), the organic-rich sediments are mainly present in the upper part of the sediment cores (unit III). The Ca-rich sediments occur in an intermediate stratigraphic position (unit II) and overlie late-glacial clayey silts (unit I).

We identified 14 seismostratigraphic event horizons (EH) on which several mass-transport deposits (MTDs) are located, indicating coeval failures of multiple subaqueous slopes (EHs A-O; Fig. 4; Supplementary Figs. 1–4; see also Daxer et al.^20^). The stratigraphic positions of the lowermost four EHs L-O are not within the studied interval of the sediment cores.

The uppermost ~ 11 m of the sedimentary infill of the western basin in core WOER18-L5, which is representative of the sedimentary succession in all lake basins, mainly consist of (from top to bottom) (1) faintly to non-laminated, calcite-poor, organic-rich silt comprising the last ~ 2.8 ka (herein called unit III; 0–1.75 m); (2) finely laminated calcite-rich silt spanning from ~ 2.8 to 13.7 ka (unit II; 1.75–5.3 m) and (3) grey clayey silt denoting the Late Glacial period older than 13.7 ka^48^ (unit I, 5.3–11 m; Fig. 4, Supplementary Fig. 6). The base of the cored interval in this study is marked by a several m thick MTD and associated megaturbidite^48^ (Fig. 4). Turbidites of strongly varying thicknesses (0.2 to ~ 30 cm) are frequently intercalated in the background sediments (Fig. 4, Supplementary Figs. 7–15). Of the event horizons within the studied interval, all but EH-F and EH-I can be reliably correlated to some of the thicker turbidites (Supplementary Fig. 16).

Within the studied interval comprising the last ~ 14,000 years, 90 events are present. Of these, 4 events have a very high, 8 have a high, 32 have an intermediate, 23 have a low and 23 events have a very low confidence level (Fig. 4). Hereinafter, the 44 events with an intermediate or higher confidence level will be referred to as earthquakes, as they meet the synchronicity criterion.

**Figure 5:**
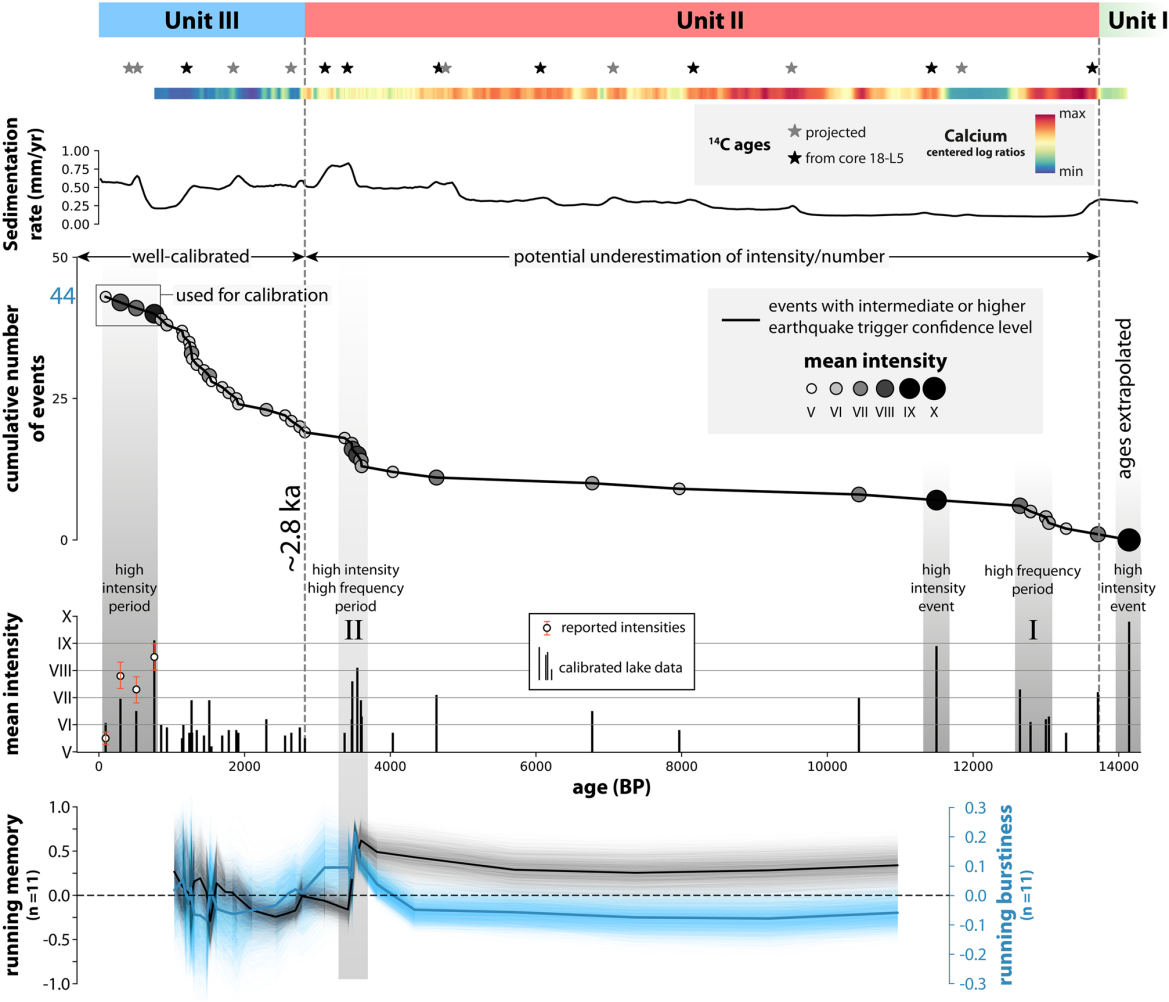
Temporal distribution of recorded events in Wörthersee. The sedimentation rates derived from the age-depth models show a general increase towards the core top. Three stratigraphic units are identified by sedimentological and geochemical data. 44 event deposits of intermediate and higher earthquake triggering confidence level are recorded. Their temporal distribution shows two phases of increased earthquake frequency (marked I and II) and several high intensity events/periods. The running average (N = 11) of the memory coefficient and the burstiness is around zero in unit III, whereas it diverges from zero in unit II. As a measure of uncertainty, the running memory and burstiness of all age model iterations are plotted in semi-transparent grey and blue lines, respectively.

Few earthquakes (19) are recorded during the interval from ~ 2.8 to 14 ka BP, except for two intervals of temporally clustered earthquake occurrence at ca. 12.8 ka BP (high frequency period I; Fig. 5) and ca. 3.5 ka BP (high frequency period II). From ~ 2.8 ka BP onwards, an increased occurrence of earthquake-related event deposits can be observed. Sedimentation rate has been proposed as a main parameter governing subaquatic slope stability and therefore a lakes’ sensitivity to record earthquake shaking as mass-wasting induced turbidites^55,56^. However, no correlation between changes in sedimentation rate and event frequency can be found in our record. The only major shift in sedimentation rates (from ~ 0.25 to ~ 0.5 mm/year) occurred at about 5 ka BP—well before any rise in event frequency can be noticed. In contrast, the increase in recorded earthquakes coincides with the lithological change from stratigraphic unit II to unit III. Due to their low density and enhanced presence of air pockets (Supplementary Fig. 6), organic-rich sediments are relatively more prone to resuspension and erosion^57^, whereas increased carbonate content may increase sediment cohesion and strength^58^. We therefore attribute the increase of recorded earthquakes in the organic-rich unit III to a lowering of the EQRT compared to the calcite-rich unit II, rather than an increase of seismicity.

Of the 44 recorded earthquakes, 21 fall into the intensity range V ≤ I < VI, 12 into VI ≤ I < VII, 7 into VII ≤ I < VIII and 3 into VIII ≤ I < IX. However, as the EQRT is not stable throughout the cored time interval, the estimation of seismic intensities based on calibration with historical earthquake data can only be reliably applied to unit III. The intensities and/or number of recorded earthquakes in unit II are potentially underestimated due to an increased EQRT, leading to an “incomplete” record of moderate (I–V) and strong (I–VI) earthquakes before ~ 2.8 ka BP.

### Earthquake recurrence statistics

Statistical analysis was carried out on events with an intermediate or higher earthquake trigger confidence level. Due to the presumed change in EQRT at ~ 2.8 ka BP, we treat units II and III separately and discard unit I because of the insufficient number of events (1).

**Figure 6:**
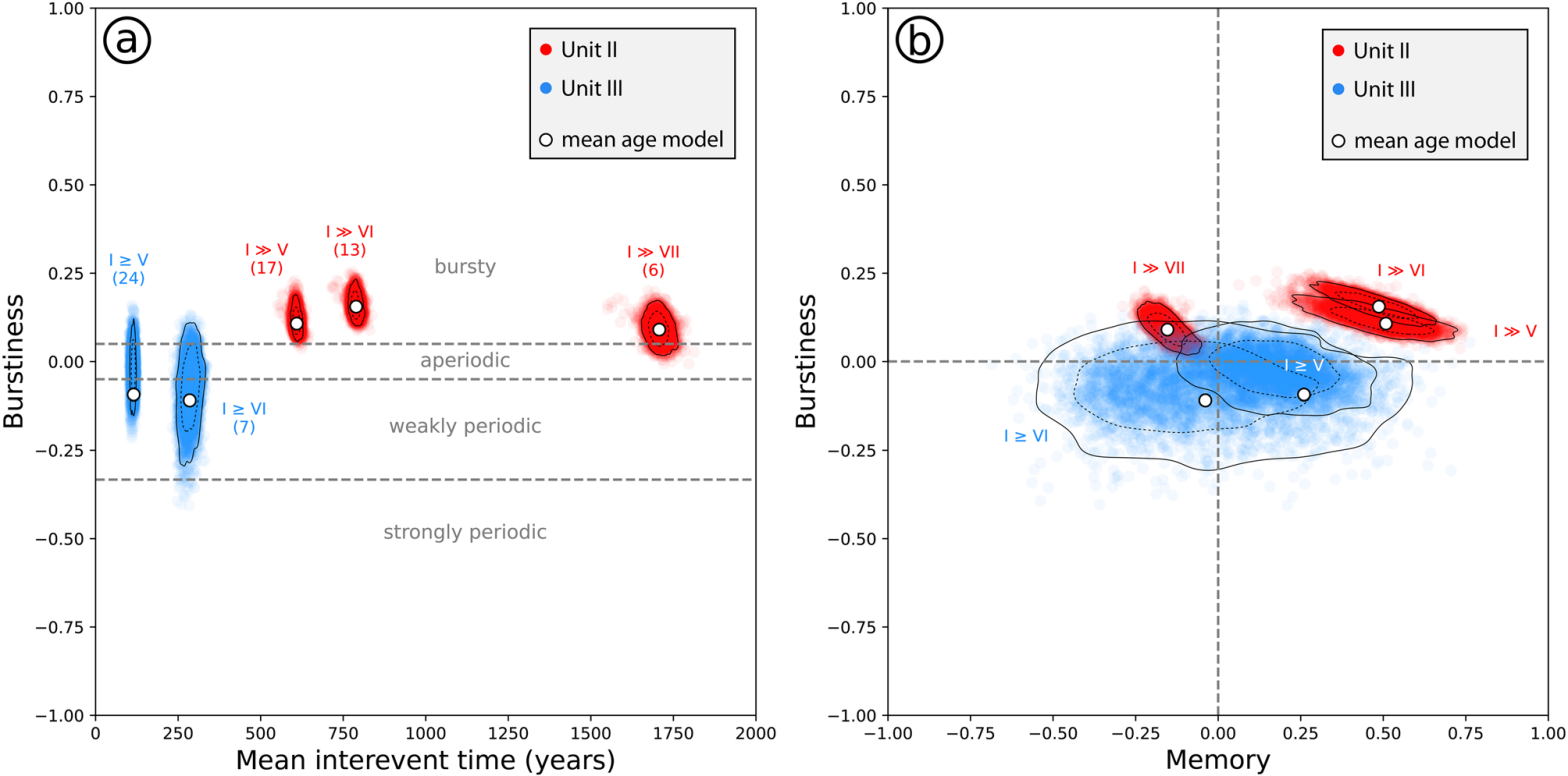
(**a**) Mean interevent time vs. burstiness diagram and (**b**) memory coefficient vs. burstiness diagram of different subsets of the turbidite paleoseismic record from Wörthersee. Individual datapoints result from different age-depth model iterations (> 3500 in total). Solid lines indicate 95% confidence intervals, dashed lines 68% confidence intervals. Numbers in brackets indicate the number of interevent times. In unit II, event numbers and/or intensity are underestimated due to the higher EQRT compared to unit III.

In unit II, interevent time for I ≥ V averages at 608 ± 755 years (mean ± standard deviation of interevent times) and at 788 ± 1078 years for I ≥ VI (Fig. 6; Supplementary Table 4). The Gamma distribution with a shape parameter α < 1 shows the best fit for both intensity classes and indicates a bursty (clustered) recurrence pattern. Statistical tests suggest rejection of the exponential distribution and thus of a Poissonian process. In unit III, the average interevent time is 114 ± 94 years (I ≥ V) and the statistical tests do not allow rejection of the exponential distribution. Together with the best fitting distribution (Gamma with α = 1.45; see also Supplementary Fig. 17), this suggests an aperiodic (Poissonian) or weakly periodic recurrence pattern.

For the lower intensity classes (I > V and > VI), the 95% confidence intervals of the unit II datasets confirm the bursty character and a positive M. For the intensity class > VII, an aperiodic or bursty behaviour and a slightly negative M can be inferred. The small number of interevent times (N = 6) of this class, however, strongly affects the reliability of the B and M values, which therefore should not be interpreted. For the unit III datasets, an aperiodic or weakly periodic recurrence pattern is confirmed, but due to shorter interevent times, the effect of age uncertainty on B is larger (~ 18% and ~ 31% for the I ≥ V and I ≥ VI subsets, respectively). M is slightly positive for the I ≥ V subset, and scatters around 0 for the I ≥ VI subset, as does the running M (N = 11; Fig. 5). Because the number of interevent times is rather large in unit III (24), age uncertainty plays a smaller role^13^. The shortest interevent times found in unit III are 8 years, thus supporting the assumption that the lacustrine paleoseismic record only contains mainshocks. Given this and the record’s agreement with a Poissonian behaviour, the well-calibrated unit III, I ≥ V subset is comparable to the input data of PSHA in Austria and thus suited for the testing of the seismic hazard curve at Wörthersee.

## Discussion

### Testing seismic hazard curves with lacustrine paleoseismic records

**Figure 7:**
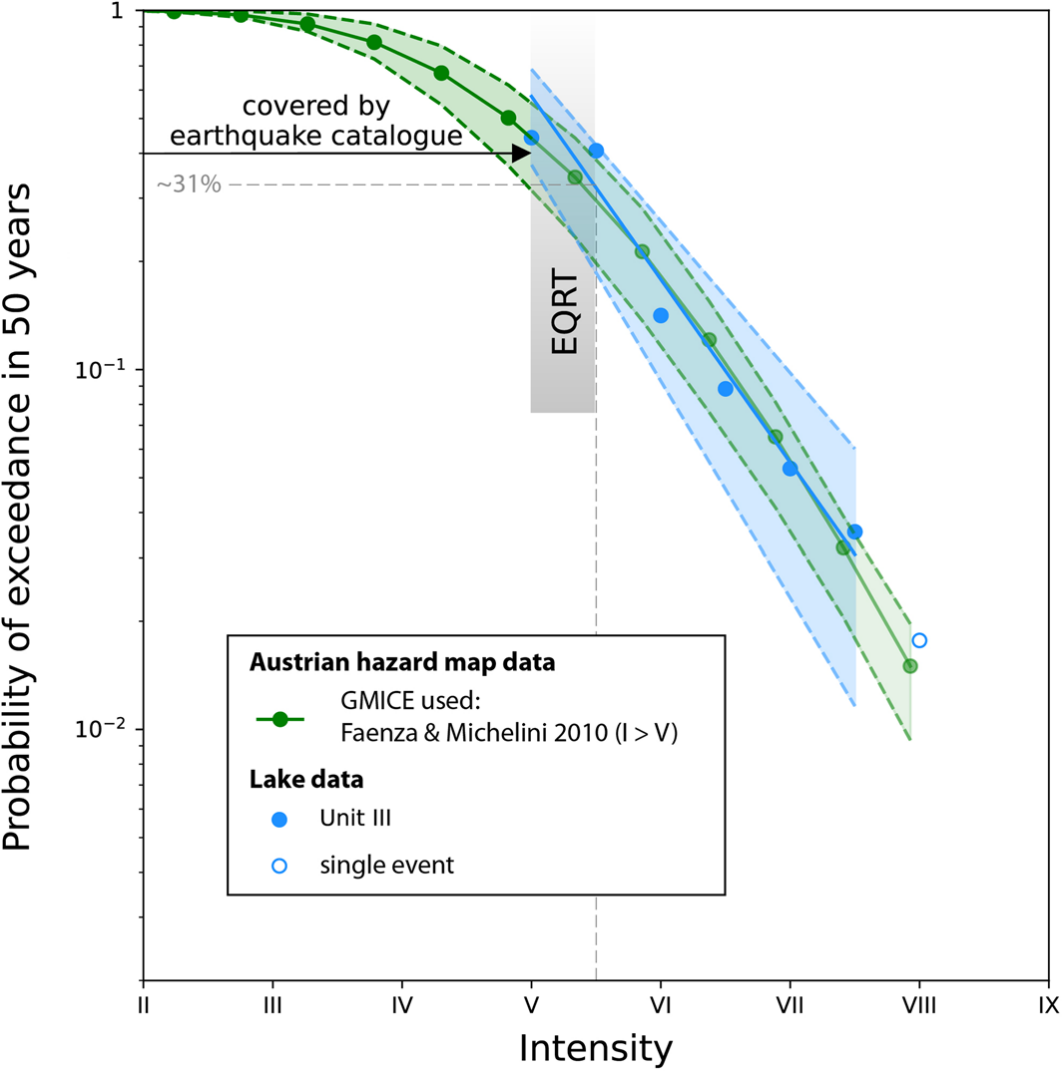
Comparison between the seismic hazard curves derived from lacustrine data of unit III (blue) and the probabilistic seismic hazard analysis based on instrumental seismicity^29^. An exponential fit, i.e. a visually linear decrease when exceedance probability is plotted on a logarithmic scale, is found for the probability of exceedance of certain intensities in 50 years for the lake dataset. The boundaries of the uncertainty range of the lake data (light blue) represent the hazard curves if I = I_min_ for the lower boundary and I = I_max_ for the upper boundary (see Fig. 3). For the PSHA data, the uncertainty range (light green) is bounded by the 16% and 84% quartiles of the probabilities derived from all logic tree branches. Additional—but hardly quantifiable—uncertainty arises from usage of a GMICE for the conversion of PGA to macroseismic intensities.

To construct a seismic hazard curve based on our lacustrine data, we calculated the exceedance probability in 50 years (PoE_50_) of a certain intensity I_x_ by dividing the number of recorded events (I ≥ I_x_) by the studied record span and multiplying by 50. The data show that PoE_50_ decreases exponentially with seismic intensity (Fig. 7). For the well-calibrated unit III, there is a ~ 31% probability of an I ≥ V½ event in a period of 50 years.

To test the seismic hazard curve of the Wörthersee area (site 46.623° N, 14.158° E) provided by ZAMG^29^, we converted the PGA values of the seismic hazard curve to intensity degrees using the ground motion to intensity conversion formula (GMICE) given by Faenza and Michelini (2010; FM10; using the coefficients of the double-line regression applicable to I ≥ V)^59^ and compared it with the PoE_50_ obtained from lake data (Fig. 7). The seismic rates in Carinthia are based on a catalogue of earthquakes which only generated I ≤  ~ V at Wörthersee. The lake data provides information on events with I ~ V–VIII and therefore constitutes an invaluable addition to the instrumental and historical dataset considered by the probabilistic approach. Our data show a very good agreement between both datasets, thus verifying the validity of the probabilistic seismic hazard analysis and the underlying models in the area of Wörthersee.

Potential uncertainties arise from the transformation of PGA (the PSHA outcome) into macroseismic intensities. Applying different GMICEs developed locally for the area of Switzerland^60^ or based on global compilations^61^ would move the seismicity-based hazard curve to lower left (lower hazard) and our lake data would suggest a higher hazard than on the seismic hazard map. However, FM10^59^ is also used to model the impact of large earthquakes in neighbouring countries as shakemaps^39^ and agrees with instrumentally-recorded PGA and documented macroseismic intensity in Austria for smaller intensity values. Altogether, we consider FM10 the most appropriate choice for our study area. Moreover, local parameters such as the presence of a sedimentary cover, bedrock slope and topographical irregularities can modify the amplitude, duration, and frequency-content of ground motion. For the comparison of lacustrine vs. probabilistic seismic hazard curve, we have used the original outcome of the PSHA which is related to soil class A (rock, VS30 > 800 m/s). As the intensity data points from historical earthquakes used for the calibration of the lacustrine sediments^20^ stem either from locations on rock or very dense sand or gravel, we consider this a reasonable assumption for our first-order approach. It is important to note that all mentioned intensity values relate to the lake surroundings and do not represent the level of ground motion on the lake bottom or slopes.

### Implications for seismic hazard analysis

Lake paleoseismology provides independent data for testing a hazard map at one specific location. Since the output of PSHA is a collection of hazard curves at specific sites without spatial correlation, testing on a site level is the recommended approach by the seismologic community^9,18^. However, due to the very long time (especially in low-seismicity regions) required to observe a sufficient number of earthquakes at a site^58^, suitable earthquake records are rarely available. The calibrated paleoseismic record of Wörthersee provides suitable data to—for the first time ever to our knowledge—independently test the performance of seismic hazard analysis by a long-term record of seismic shaking. It confirms the applicability of time-independent probabilistic seismic hazard modelling in the area of Carinthia given the potentially Poissonian earthquake occurrence in the last ~ 2.8 ka of the record. Furthermore, the intensity-frequency relationship of earthquakes recorded in the Wörthersee sediments agrees with the seismic hazard curve, and thus validates the current PSHA approach for this part of Austria. The availability of suitable lake systems for paleoseismology in the European Alps^62,63^ provides a way forward to test the performance of national seismic hazard maps on multiple sites ranging from low (10% PoE_50_ of ~ 0.5 m/s^2^ in the Alpine foreland) to high hazards (10% PoE_50_ of ~ 2.5 m/s^2^ in the Friuli/Slovenia region). Together with other types of paleoseismic data, the lacustrine records also help to constrain the timing of specific paleo-earthquakes and to reconstruct plausible combinations of magnitudes and epicentre locations^62,63^. For instance, the earthquake recorded in Wörthersee at 10,966–9950 BP could potentially correspond to fault slip archived in speleothems of the Obir caves between 10,730 ± 230 and 8610 ± 150 BP^64^. A M_w_ of 5.5 and epicentral intensities of VIII to IX were proposed for this event^64^. Given the seismic intensity of ~ VII (or higher) at Wörthersee and typical intensity attenuation rates in the Alps^65^, this would imply a M_w_ of ~ 6–6.5 in case these paleoseismic data correlate. Although the age overlap is small, another speleothem damage dated between 6280 ± 240 and 5700 ± 1200 BP^64^ might correspond to the earthquake recorded in Wörthersee at 4767–4523 BP.

In other seismotectonic settings, i.e. at plate boundaries, it has been shown that frequency-magnitude data from instrumental seismicity may be offset from those of lake paleoseismic records^66^, because small-scale recent seismicity may not be representative for the seismic cycle of elastic strain build-up and release on major plate boundary faults. This highlights the importance of comparing (primary and secondary) paleoseismic data with instrumental seismicity data to obtain accurate input parameters for PSHA, by (1) testing the high-magnitude part of Gutenberg-Richter relationships, either for regional seismicity or individual faults, (2) constraining the maximum credible earthquake and (3) assessing the temporal distribution and recurrence pattern of large earthquakes.

Our lacustrine data, for instance, reveal non-stationarity during unit II and the possibility of seismic bursts (Fig. 5). Large positive Memory coefficients (~ 0.5) imply a tendency for short (long) interseismic time following short (long) interseismic time, and thus the bracketing of periods with different paleo-seismicity rate and behaviour. The high-frequency periods lead to exceptionally high values of B during unit II compared to most other lacustrine paleoseismic records worldwide (Moernaut^67^ and references therein). During the high frequency periods recorded in Wörthersee, the interevent times of two events with I > VI can be as short as ~ 8 years. More recent regional earthquake bursts are reported from the Austrian federal state of Tyrol^68^, with two events with I_0_ VIII and VII–VIII in 1670 and 1689, and the Italian region of Friuli^69^, where three main shocks of M_w_ 6.4, 6.1 and 5.3 occurred within 16 months in 1976–1977. Together with the identification of low paleo-seismicity periods (e.g. 5–11 ka BP) in the Wörthersee record, this suggests that seismic hazard in the Eastern European Alps might not always be constant, as assumed in classical PSHA, but can episodically increase. Incorporating such non-stationarity in the PSHA framework for Austria and its surrounding regions poses an important challenge to the hazard modelling community.

The causes of bursty lacustrine paleoseismic records can be manyfold. In slowly deforming regions, deformation is typically accommodated within a complex network of interacting faults^10^. Hence, a large area can be subject to near-critical stress conditions and small perturbations (i.e. earthquakes) have the potential to initiate rupture cascades, leading to regional seismic bursts separated by long, relatively quiescent intervals^70^. Depending on a lakes’ sensitivity to record seismic shaking, the potential source area of earthquakes that are archived in the lake sediments varies. A relatively high EQRT—as observed in Wörthersee before ~ 2.8 ka—restricts the sources contributing to the paleoseismic record to a few local, potentially interdependent faults, leading to a bursty lacustrine record with a highly positive memory coefficient. The more aperiodic recurrence behaviour and shorter recurrence times since ~ 2.8 ka are explained by the lower EQRT, and thus the inclusion of a larger number of remote fault sources over a larger region that rupture more independently.

## Conclusions

We present an innovative method to test the validity of PSHA results using a lacustrine turbidite record. The Wörthersee record reveals 44 earthquakes over the last ~ 14 ka with local seismic intensities spanning ~ V to ~ X. The frequency-intensity curve based on the lacustrine data of the last ~ 2.8 ka validates the hazard curve of standard (time-independent) PSHA in the vicinity of Wörthersee. On longer timescales, however, clustered earthquake occurrence is observed, suggesting the need for a time-dependent component of PSHA in the Eastern Alps.

Our method provides means to evaluate the use of time-independency in hazard models and to test seismic hazard curves at specific map locations (i.e. lake sites). Because a tested magnitude-calibrated intensity prediction equation which covers strong earthquakes is not available in Austria, our approach relies on a GMICE to convert the output of standard PSHA (in PGA) to macroseismic intensity. Where possible, future work should (1) focus on multiple lake sites to assess the regional accuracy of seismic hazard maps and (2) compare the lacustrine paleoseismic data with intensity-based seismic hazard curves to reduce uncertainties. Thus, the quantitative lacustrine paleoseismic approach established in this paper can be an invaluable tool to independently test hazard maps in virtually any tectonic setting.

## Supplementary Information

Supplementary Information 1.

Supplementary Table 2.

## Data Availability

Bathymetric data (1 m) can be downloaded from the dataserver of the Austrian ministry of finances: https://www.data.gv.at/katalog/dataset/106c4955-616d-4b11-97eb-8e4a64169306. All sediment core data as well as the 3.5 kHz reflection seismic data are available on zenodo: https://doi.org/10.5281/zenodo.6479186 and https://doi.org/105281/zenodo.7308844.
