# Supplementary material for: Validation of seismic hazard curves using a calibrated 14 ka lacustrine record in the Eastern Alps, Austria
Source: Sci Rep. 2022 Nov 19;12:19943. doi: 10.1038/s41598-022-24487-w (PMC9675742; doi:10.1038/s41598-022-24487-w)
Supplement: Supplementary file 1 — Supplementary Information 1. [file 41598_2022_24487_MOESM1_ESM.pdf]

Supplementary information to:

Validation of seismic hazard curves using a calibrated 14 ka lacustrine record in the Eastern Alps, Austria

Authors:

Christoph Daxer[(1)*], Jyh-Jaan Steven Huang [(1,2)], Stefan Weginger[(3)], Michael Hilbe[(4)], Michael Strasser[(1)], Jasper Moernaut[(1)]

[(1)] Institute of Geology, University of Innsbruck (Austria)

[(2)] Institute of Oceanography, National Taiwan University (Taiwan)

[(3)] ZAMG – Zentralanstalt für Meteorologie und Geodynamik, Vienna (Austria)

[(4)] Institute of Geological Sciences and Oeschger Centre of Climate Change Research, University of Bern (Switzerland)

*Corresponding author:

Christoph Daxer

Innrain 52f

6020 Innsbruck

Austria

christoph.daxer@uibk.ac.at

Supplementary Figure 1: Maps of seismostratigraphic event horizons (1)

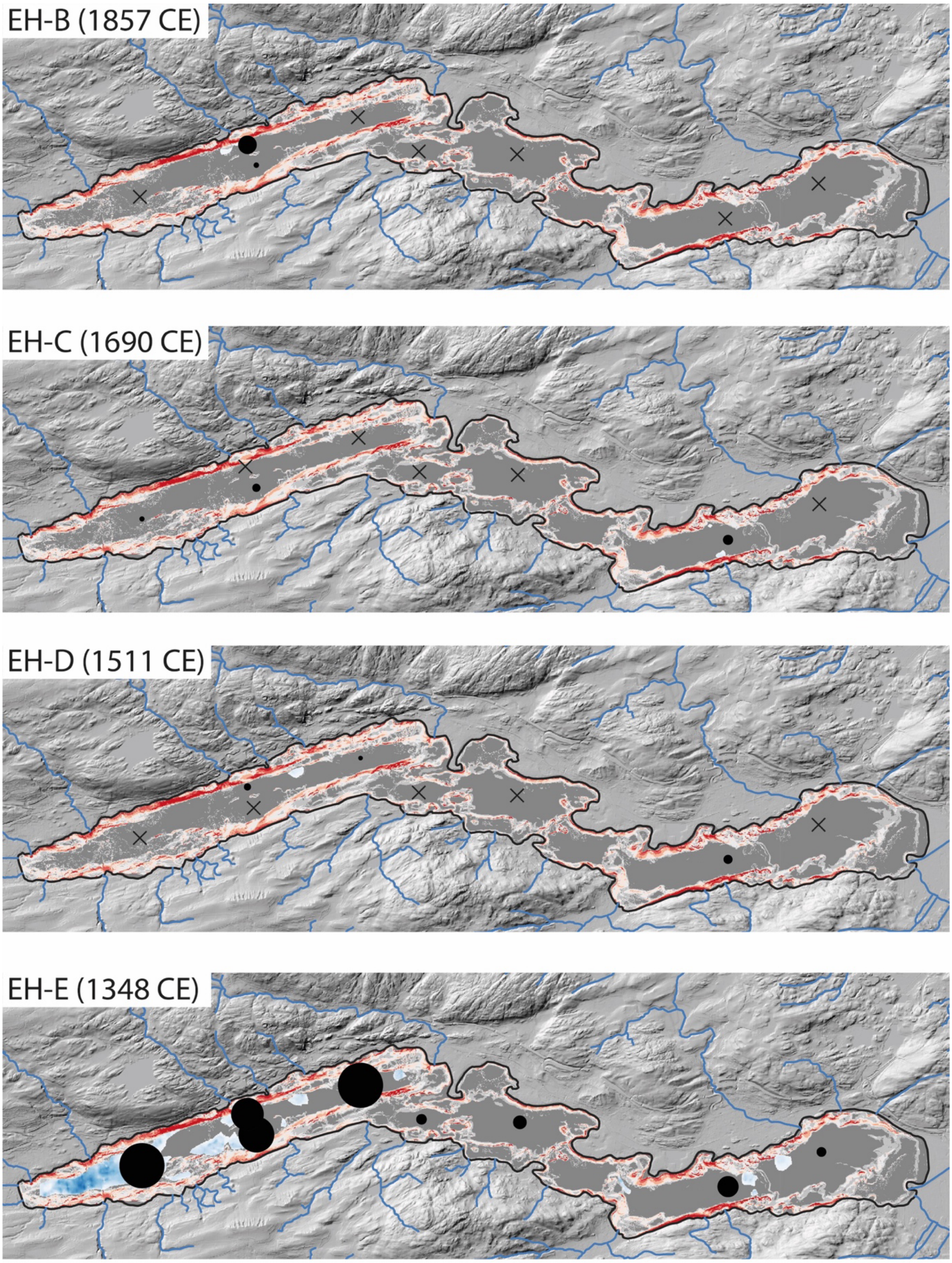

Supplementary Figure 1: Maps of seismostratigraphic event horizons and related turbidite thicknesses (for legend see Supplementary Fig. 4). The maps were generated using QGIS software (version 3.20.2; http://www.qgis.org).

Supplementary Figure 2: Maps of seismostratigraphic event horizons (2)

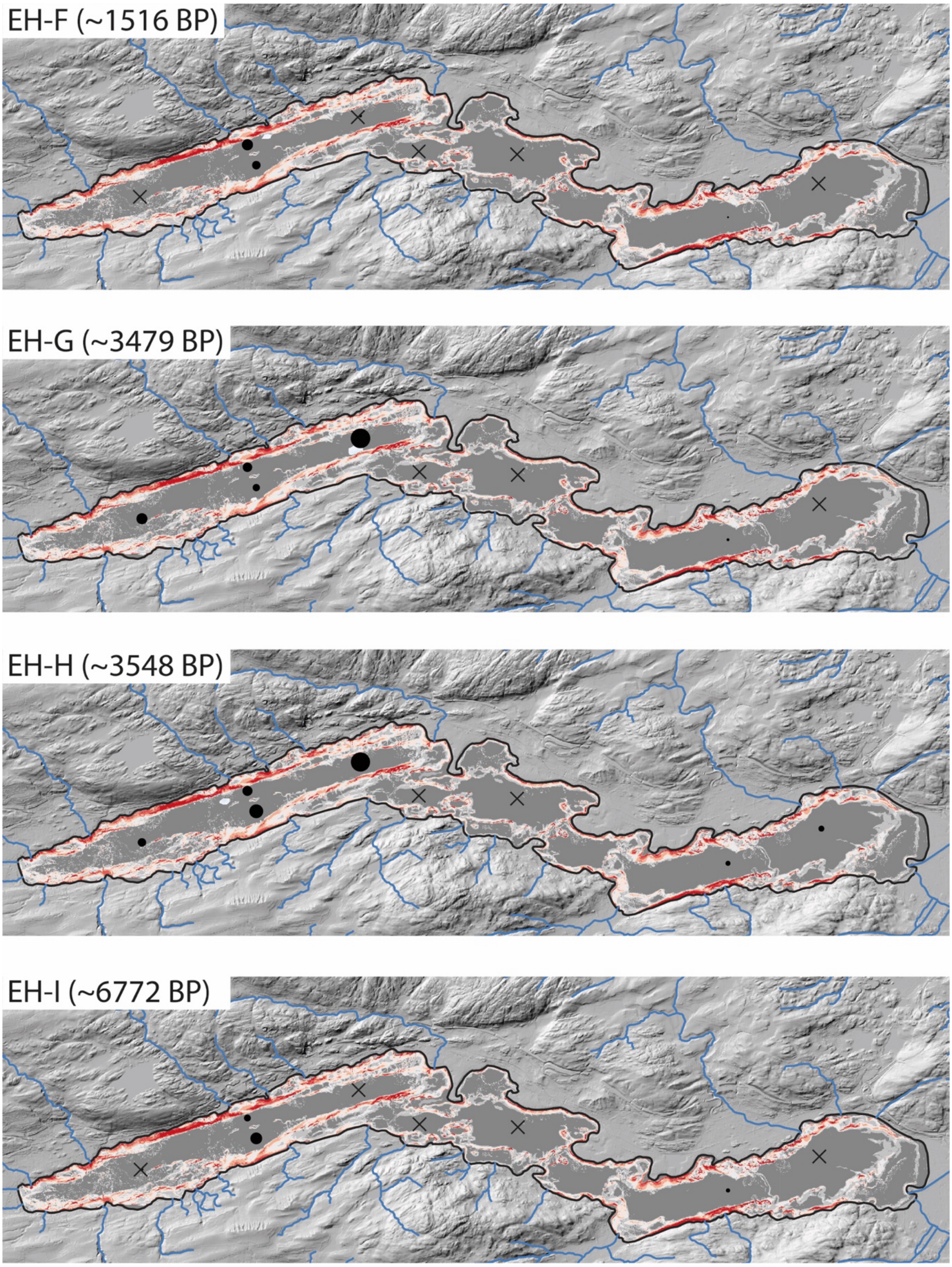

Supplementary Figure 2: Maps of seismostratigraphic event horizons and related turbidite thicknesses (for legend see Supplementary Fig. 4). The maps were generated using QGIS software (version 3.20.2; http://www.qgis.org).

Supplementary Figure 3: Maps of seismostratigraphic event horizons (3)

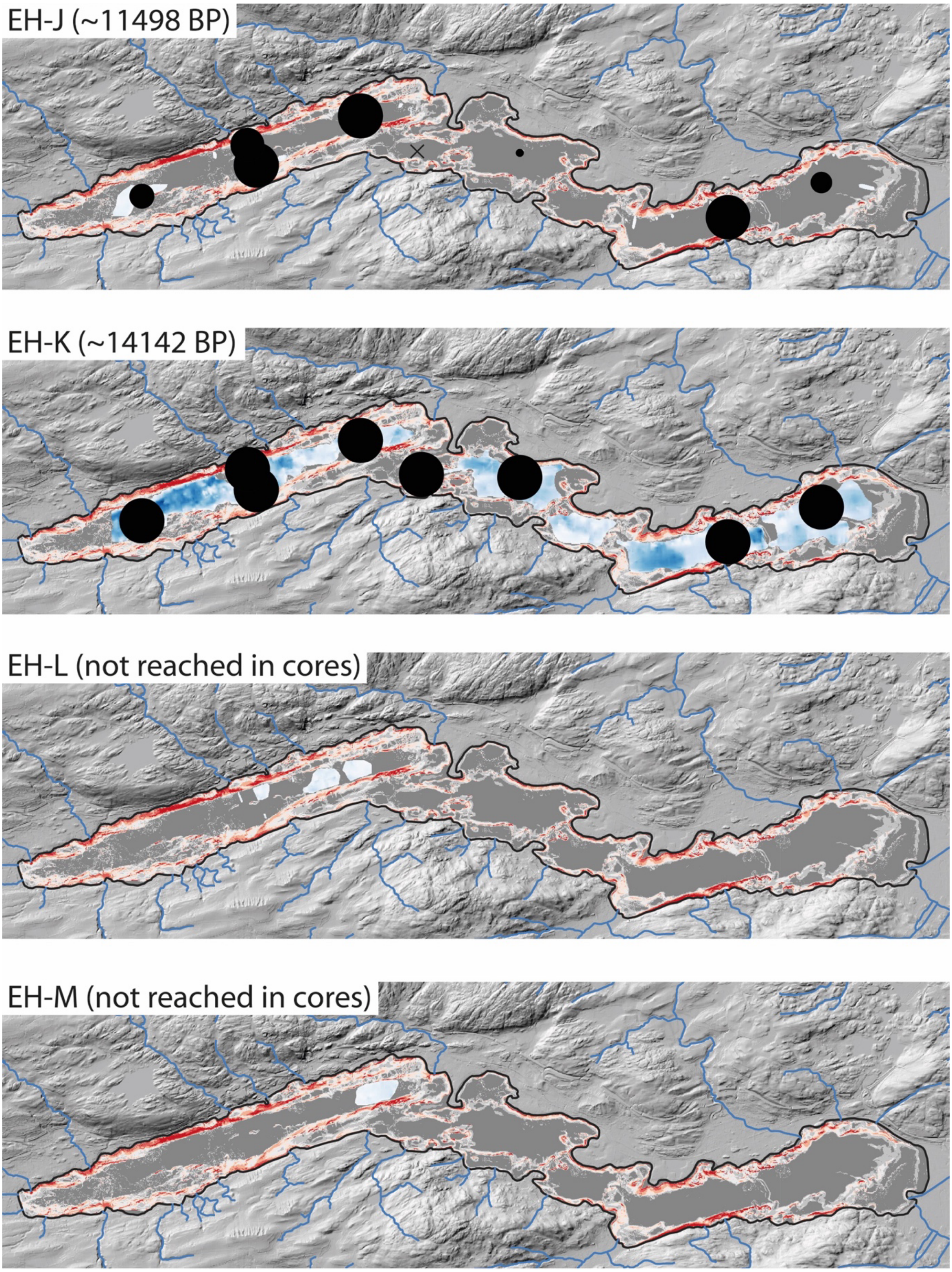

Supplementary Figure 3: Maps of seismostratigraphic event horizons and related turbidite thicknesses (for legend see Supplementary Fig. 4). The maps were generated using QGIS software (version 3.20.2; http://www.qgis.org).

Supplementary Figure 4: Maps of seismostratigraphic event horizons (4)

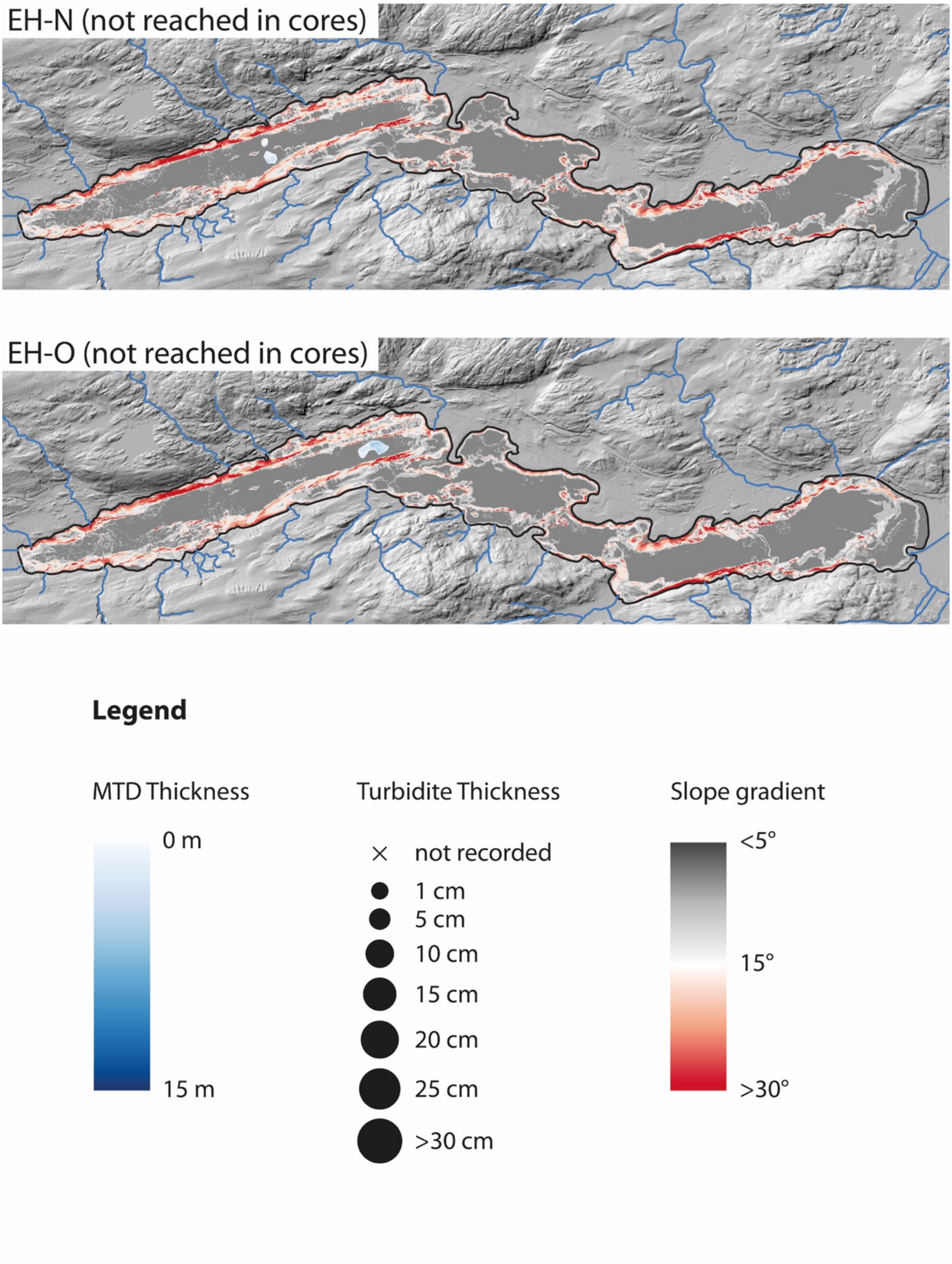

Supplementary Figure 4: Maps of seismostratigraphic event horizons and related turbidite thicknesses. The maps were generated using QGIS software (version 3.20.2; http://www.qgis.org).

Supplementary Fig. 5: Age-depth models

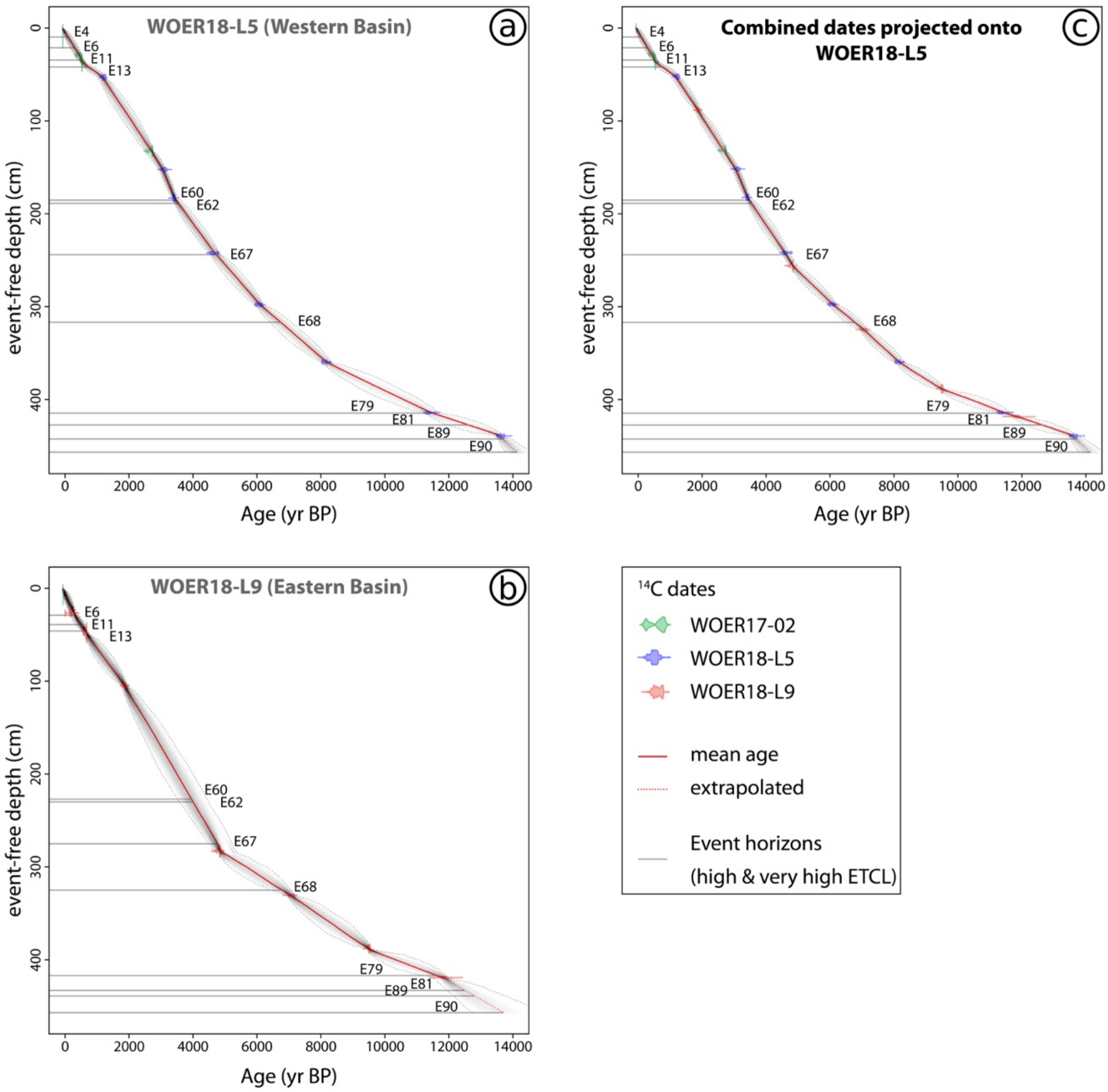

*Supplementary Figure 5: Age-depth-models. (**a**) Age-depth model of core WOER18-L5 from the western basin. (**b**) Age-depth model from core WOER18-L9 from the eastern basin. (**c**) Combined age-depth model with ages from WOER18-L9 projected onto WOER18-L5. This age-depth model is used to derive all event ages.*

Supplementary Figure 6: Lithotypes of Wörthersee

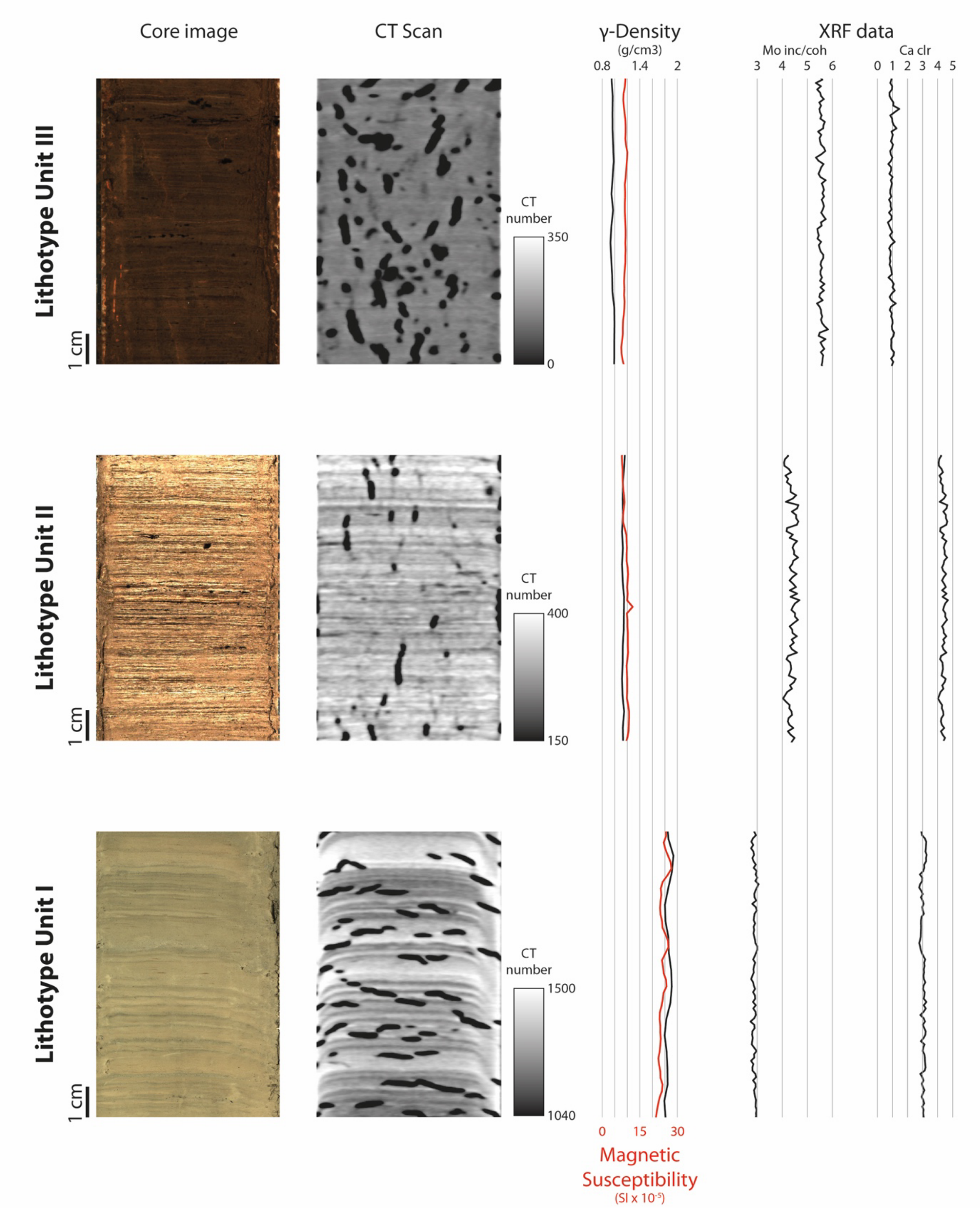

Supplementary Figure 6: Lithotypes of the Wörthersee sedimentary infill.

Supplementary Figure 7: Compilation of Turbidites

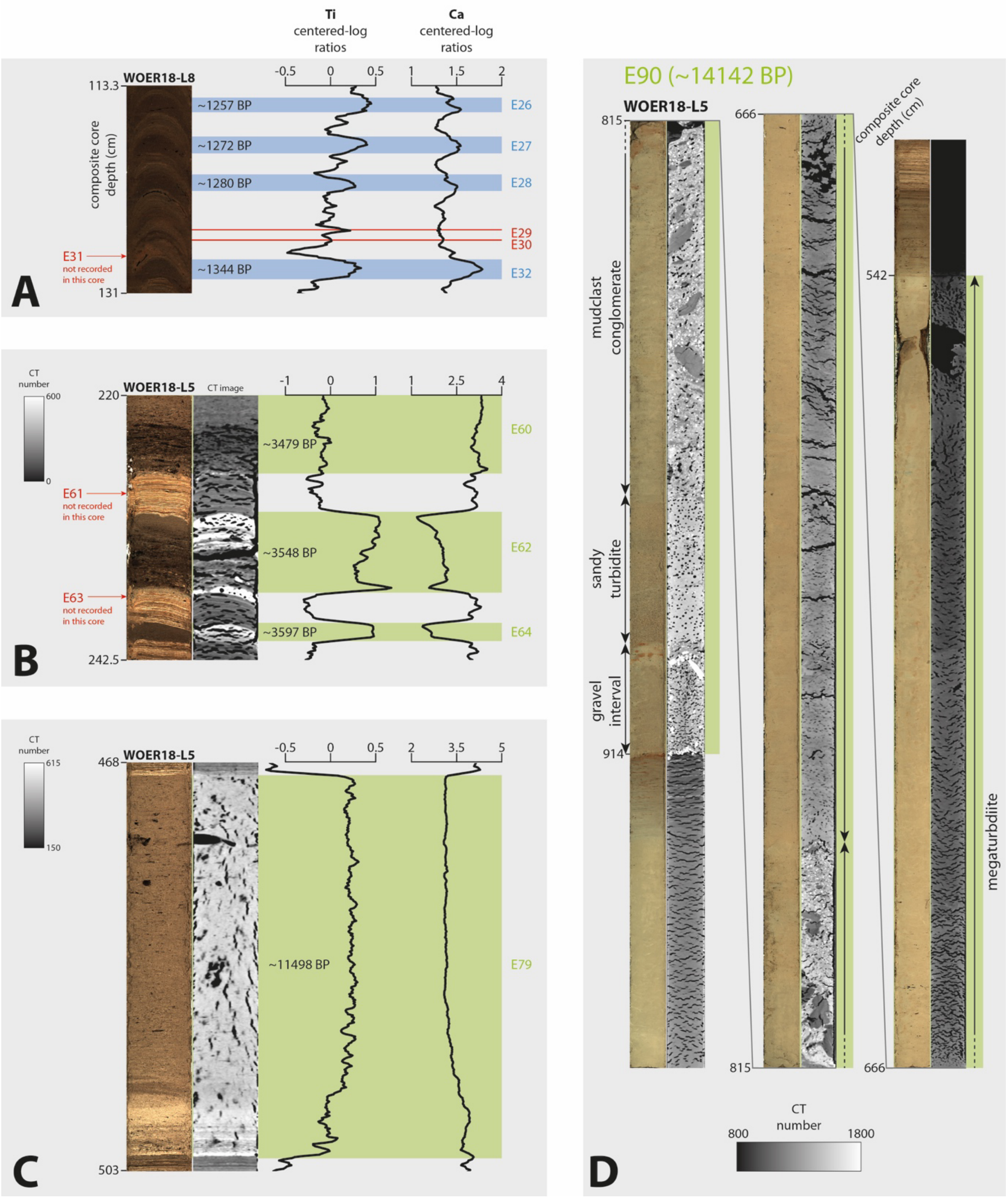

Supplementary Figure 7: A compilation of different types of turbidites recorded in Wörthersee. ***A*** *Organic-rich turbidites recorded in lithological unit III.* ***B*** *Turbidites recorded within lithological unit II (well-laminated) during high-frequency period II.* ***C*** *Thick turbidite associated with EH-J (cf. Supplementary Fig. 16).* ***D*** *Megaturbidite and underlying MTD (mudclast conglomerate) associated with EH-K (see also Daxer et al. 2020).*

Supplementary Figure 8: Core log of WOER18-L2

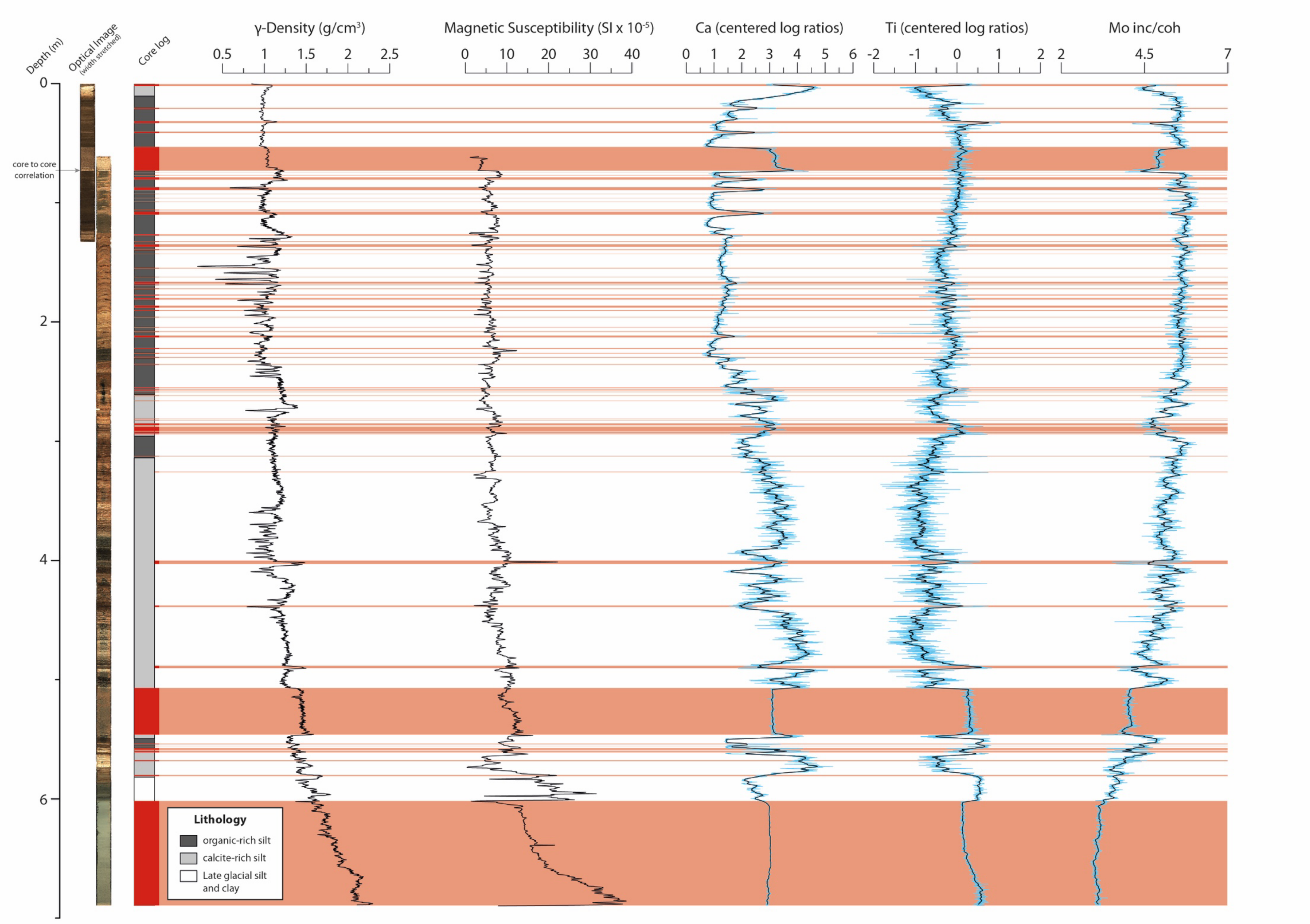

Supplementary Figure 9: Core log of WOER18-L3

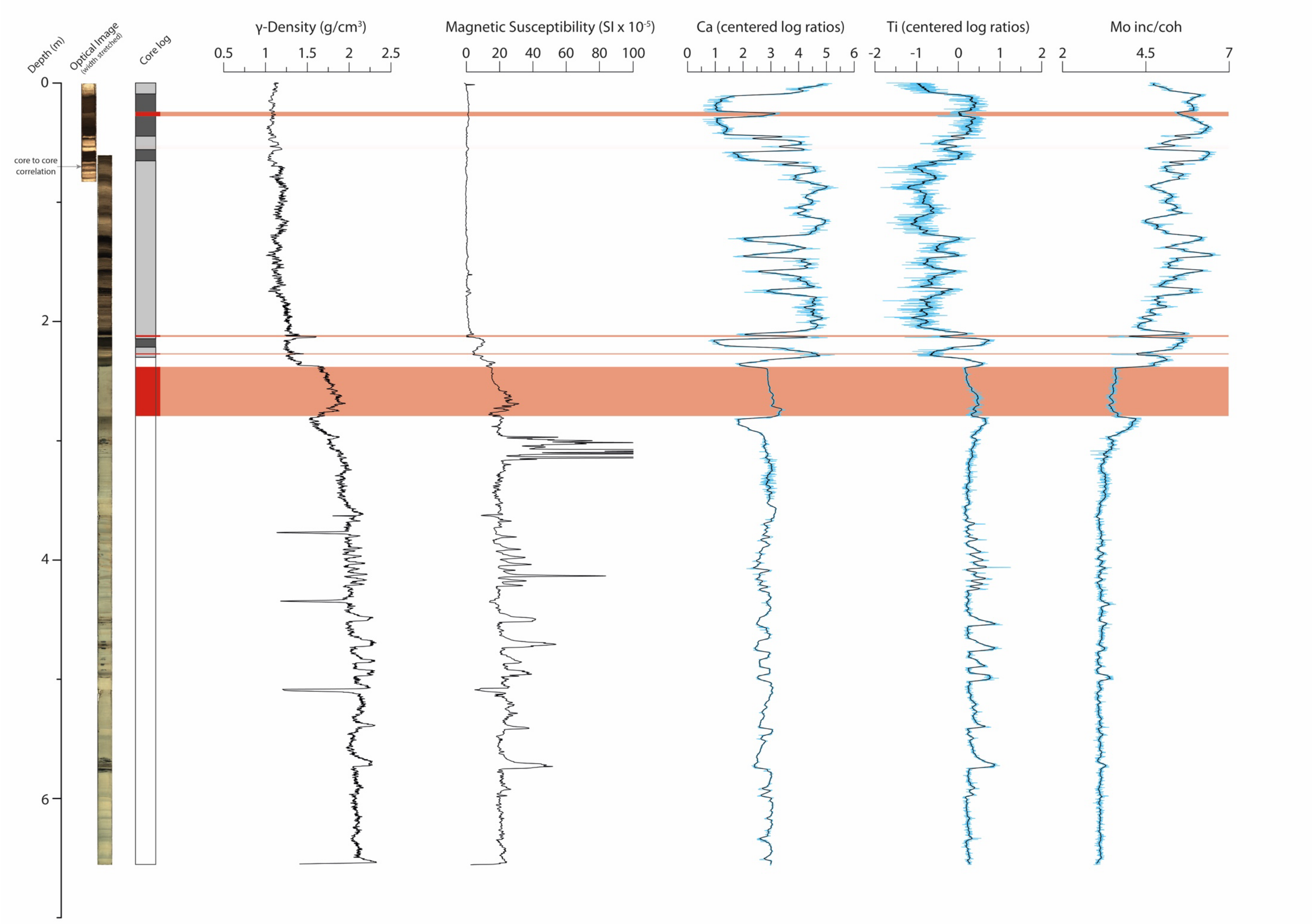

Supplementary Figure 10: Core log of WOER18-L4

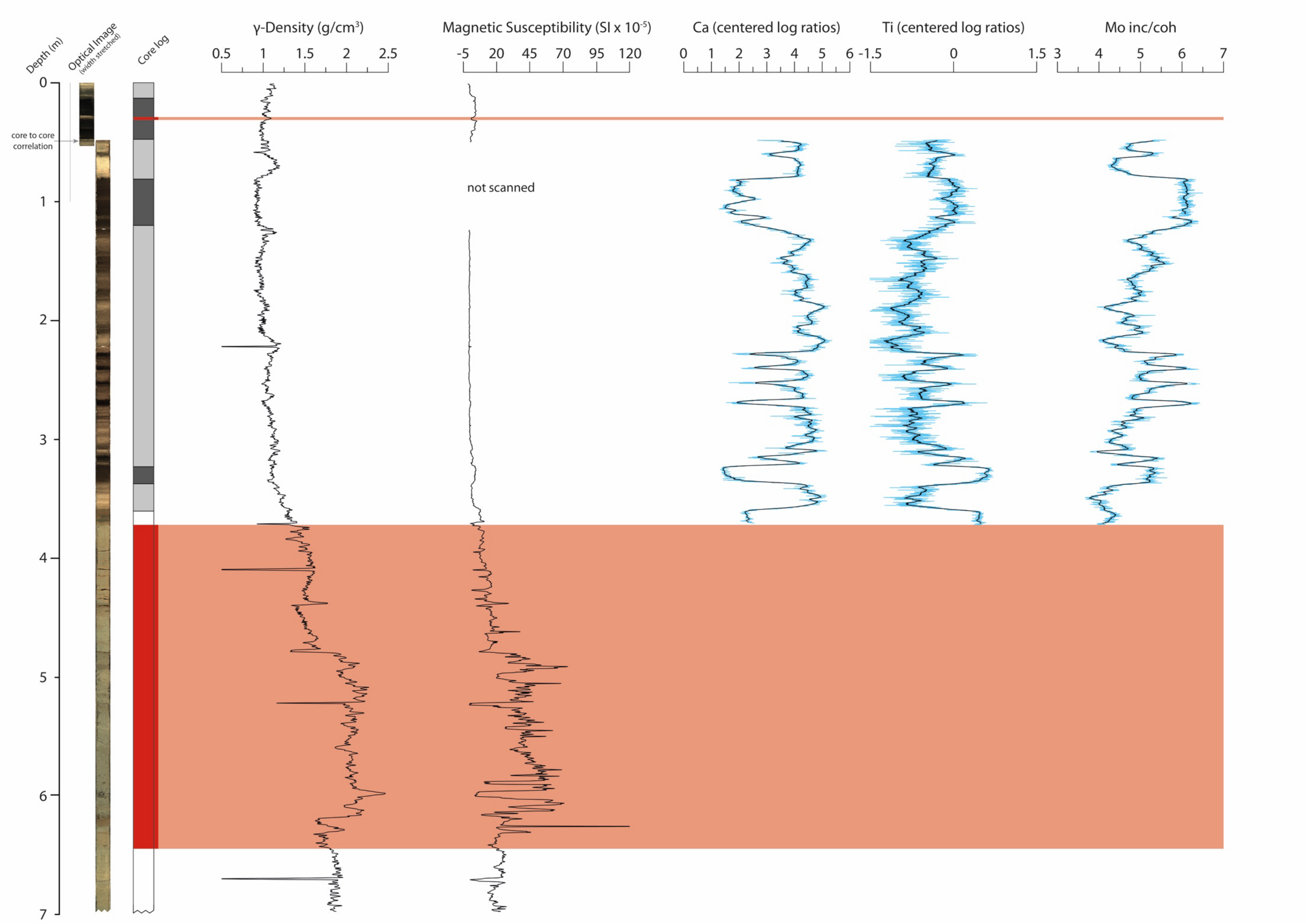

Supplementary Figure 11: Core log of WOER18-L5

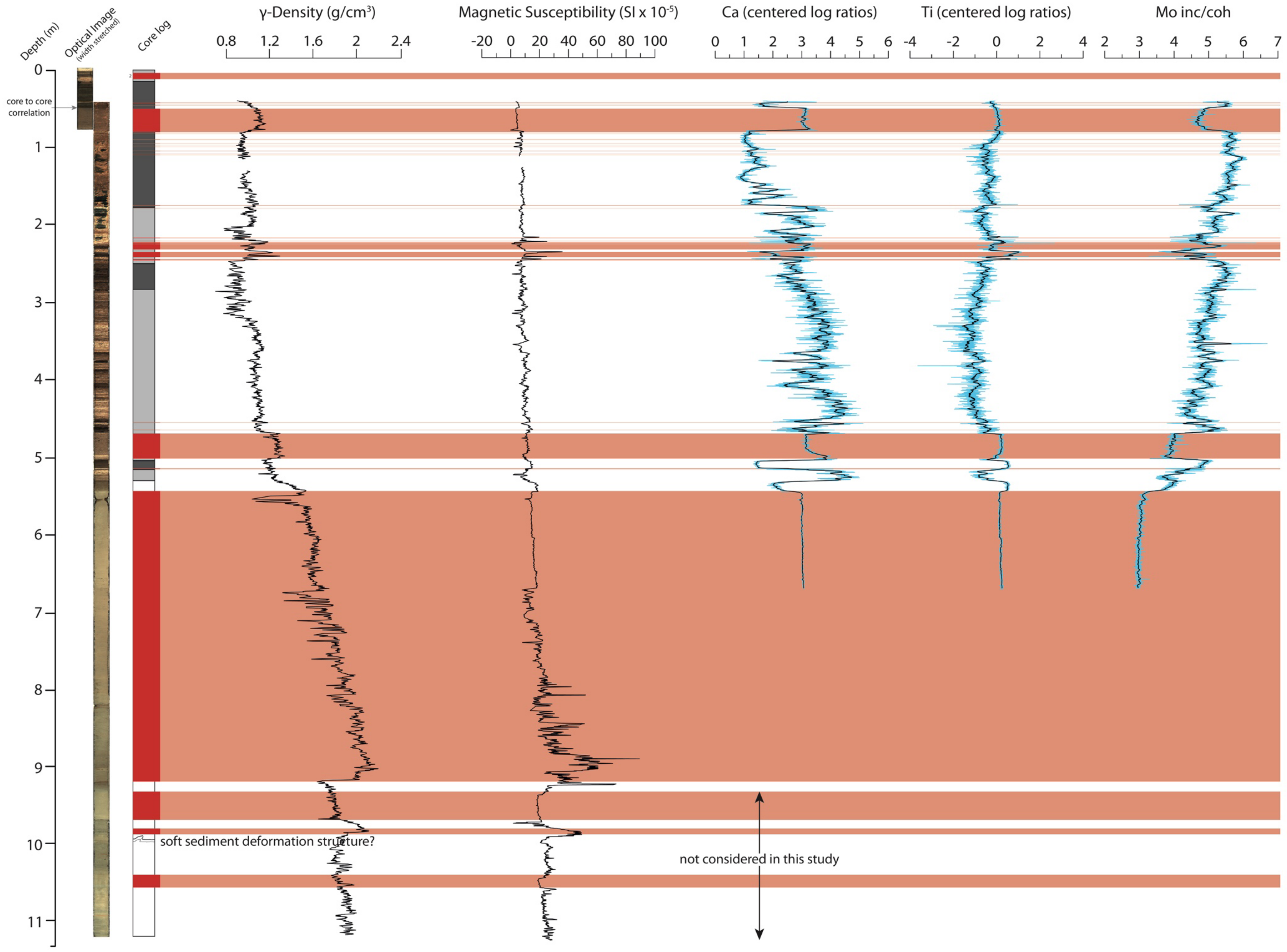

Supplementary Figure 12: Core log of WOER18-L7

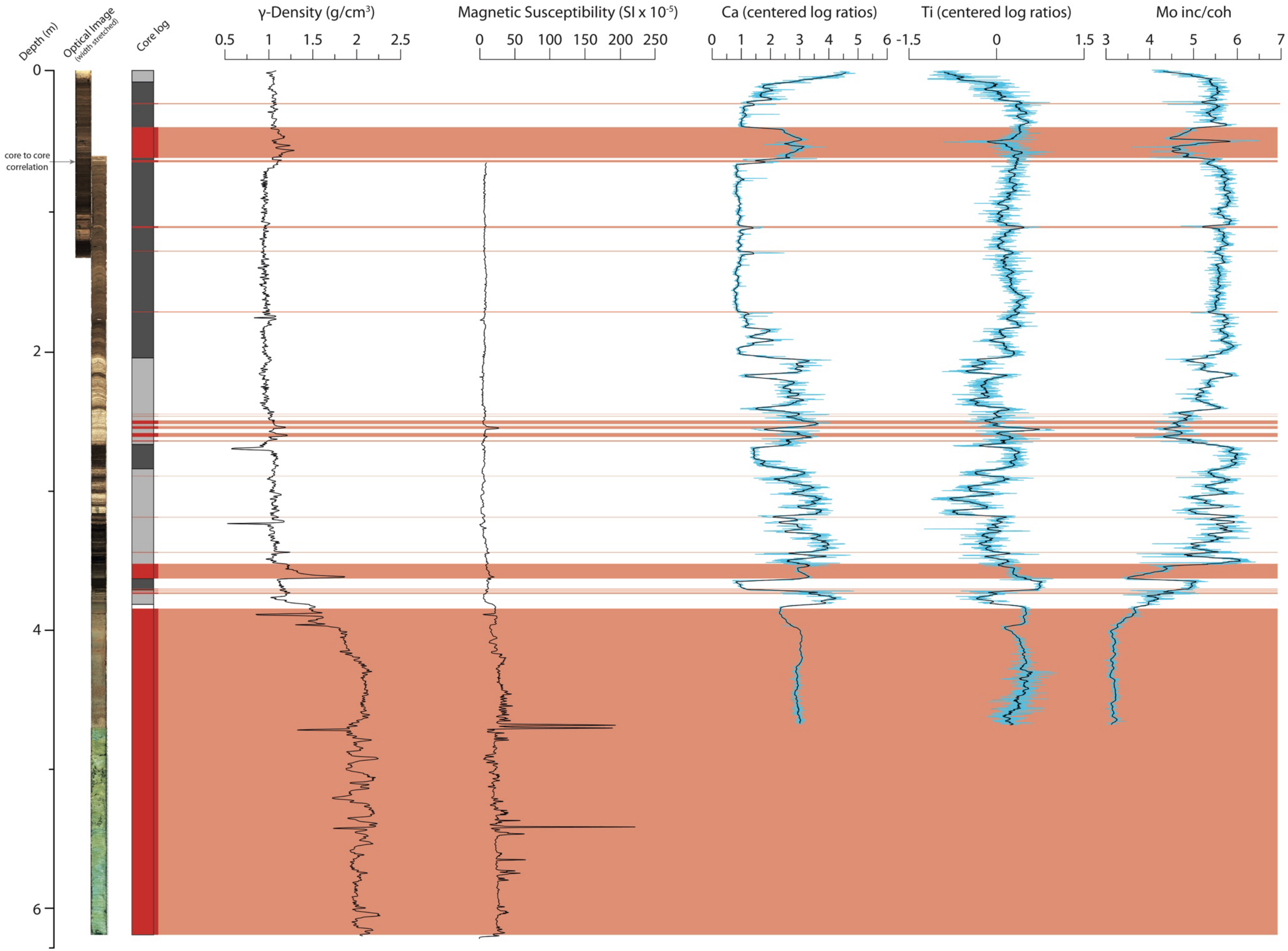

Supplementary Figure 13: Core log of WOER18-L8

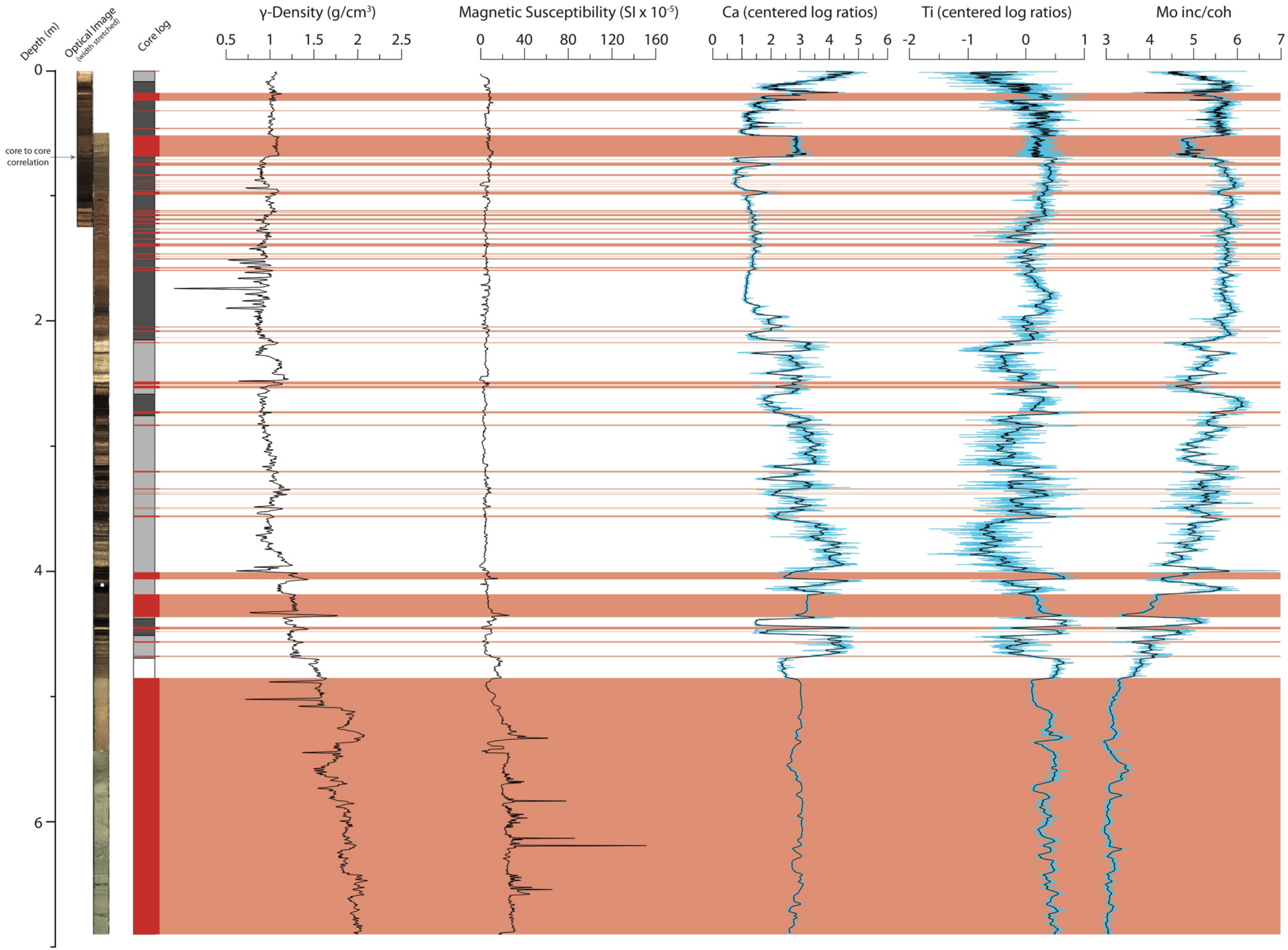

Supplementary Figure 14: Core log of WOER18-L9

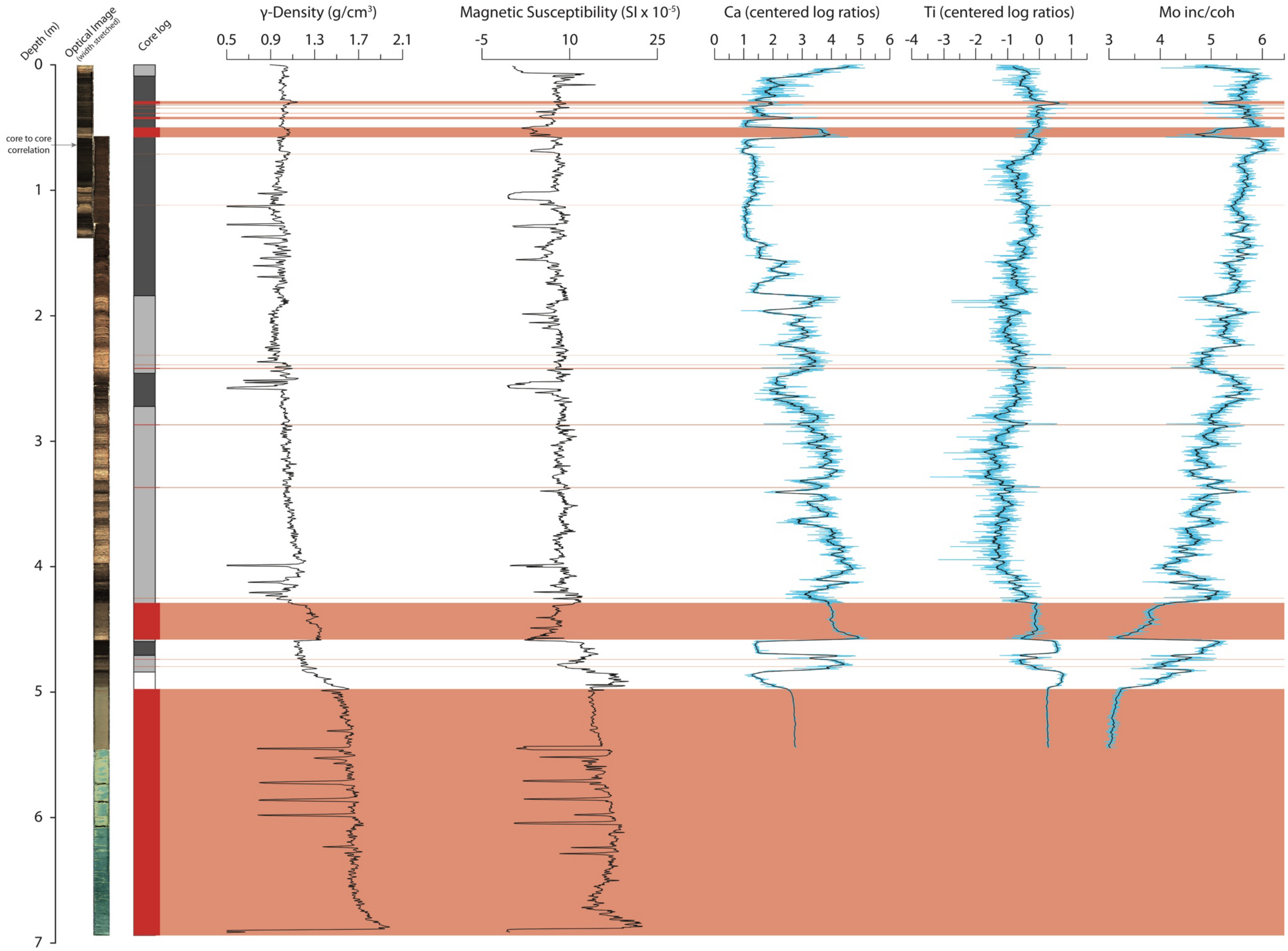

Supplementary Figure 15: Core log of WOER18-L10

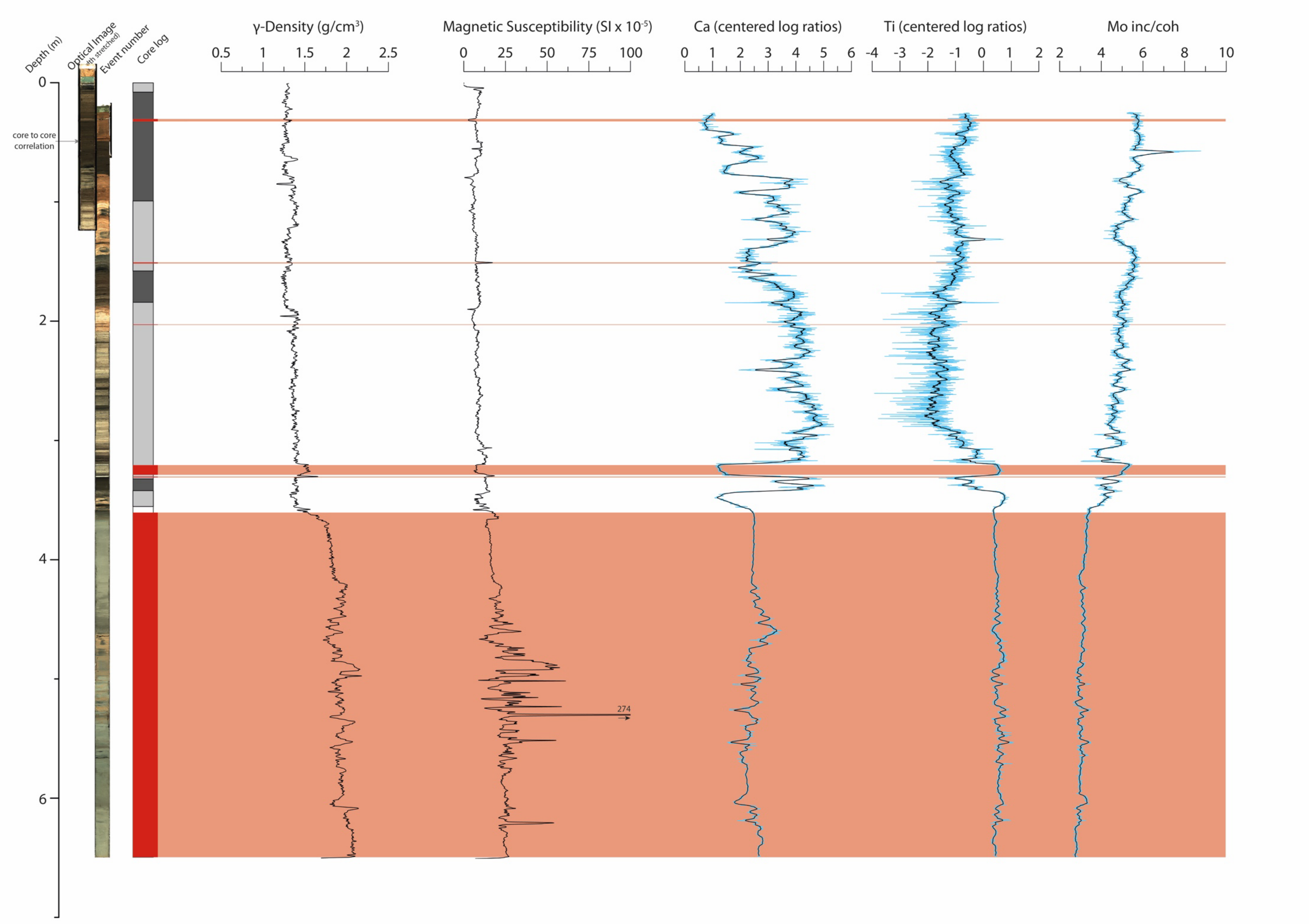

Supplementary Figure 16: Seismic-to-core correlation

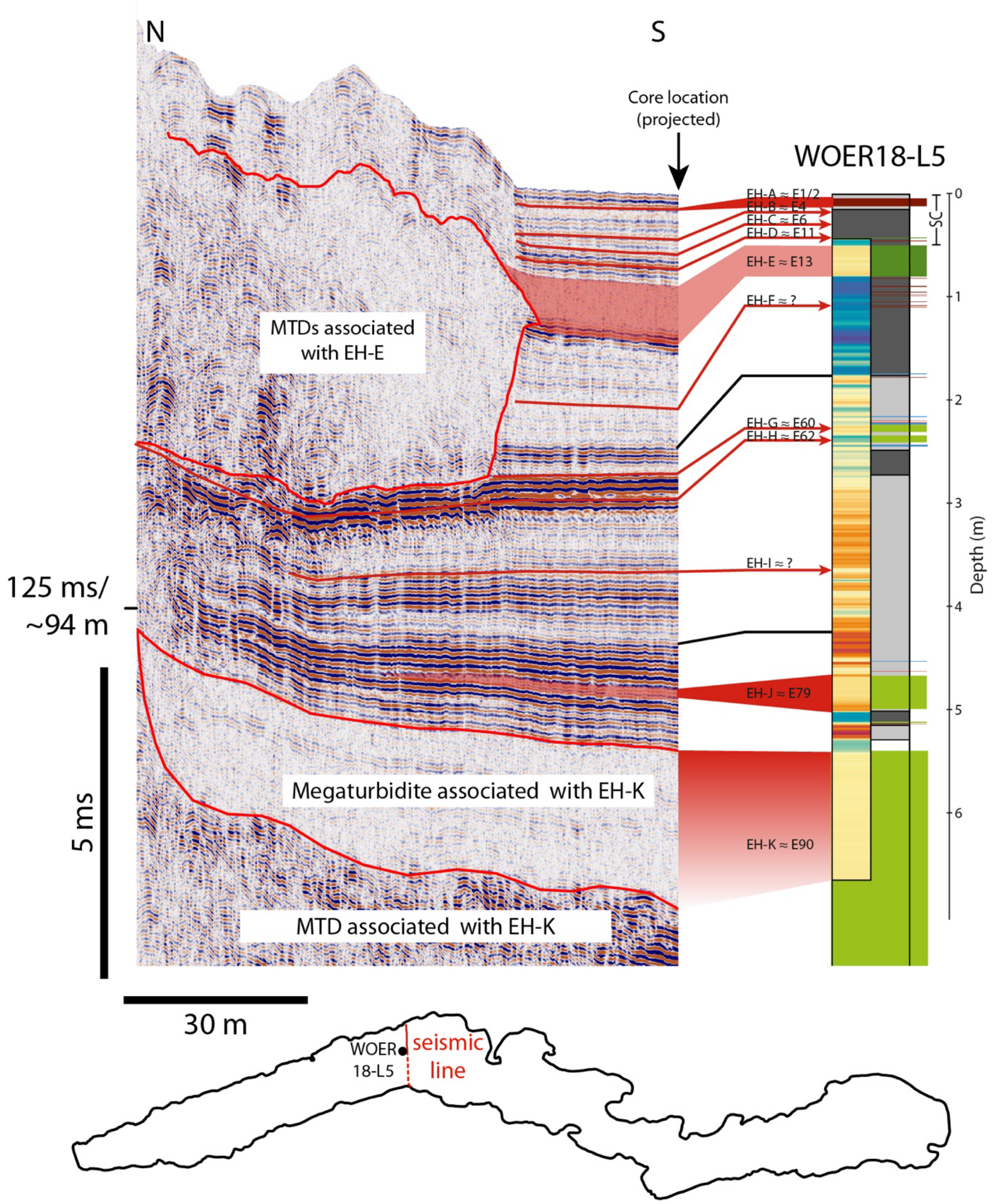

Supplementary Figure 16: Seismic-to-core correlation (8 kHz seismic profile) with indications of seismostratigraphic event horizons and their respective turbidites recorded in the sediment cores (correlated to core WOER18-L5).

Supplementary Figure 17: Interevent time statistics

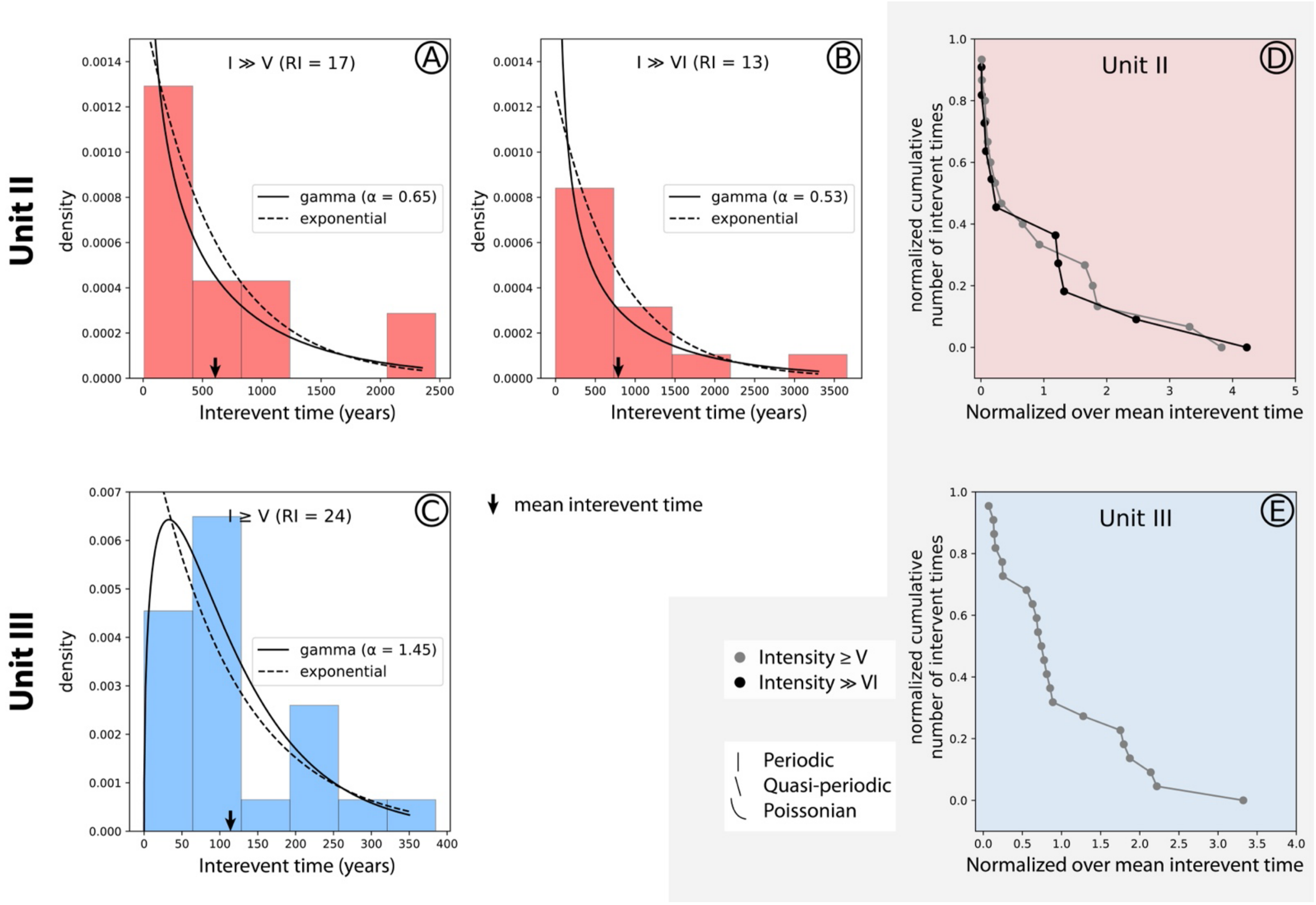

Supplementary Figure 17: Interevent time statistics of earthquakes recorded in Unit II and Unit III. ***A-C*** *Histograms of interevent times for different stratigraphic units and intensity ranges. For each histogram, the best-fitting probability distribution and the exponential distribution are indicated.* ***D & E*** *Normalized interevent data for comparison to distribution shapes.*

Supplementary Figure 18: Intensity-frequency curves of Units II and III

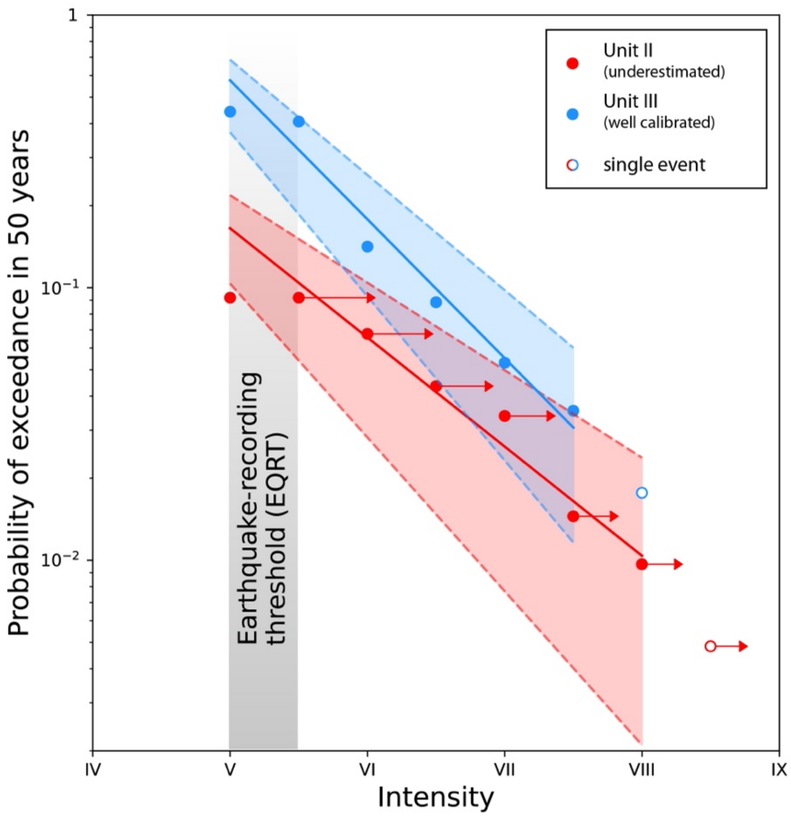

Supplementary Figure 18: Intensity-frequency-curves for paleo-earthquakes recorded in Unit II and Unit III. Although Unit II shows non-Poissonian recurrence behaviour, an exponential decrease in earthquake frequency with intensity is given.

Supplementary Table 1: Radiocarbon Ages

Core ID	Laboratory ID	Composite core depth (cm)	Event-free sediment depth (cm)	Radiocarbon age (a BP)	error (1 sigma)	95% calibrated age range (cal a BP)		Material
						from	to	
WOER17-02	ETH-88627	36.6	28.9	388	30	507	319	
WOER17-02	ETH-85092	44.6	36.9	519	23	553	509	Betula fruit, leaf remains
WOER18-L5-G	ETH-104652	90.3	52.4	1268	23	1280	1130	leaves
WOER17-02	ETH-85093	169.9	132	2562	24	2754	2522	Betula fruit, leaf remains
WOER18-L5-G	ETH-104653	189.8	151.9	2948	24	3206	3003	Pinus needle
WOER18-L5-F	ETH-104650	220.4	182.5	3184	25	3452	3368	leaves
WOER18-L5-F	ETH-104651	296.1	241.9	4103	24	4809	4523	leaves
WOER18-L5-F	ETH-96545	351.6	297.4	5273	24	6181	5941	leaf
WOER18-L5-E	ETH-104649	413.6	359.4	7355	27	8289	8031	leaves
WOER18-L5-E	ETH-96547	467.9	413.7	9952	28	11606	11256	Betula fruit, needles, leaf remains
WOER18-L5-D	ETH-96548	525.3	438.8	11785	27	13755	13519	Betula fruit, needles, leaf remains
WOER18-L9-SC_34.5cm	ETH-105423	26.5	26.5	214	21	305	…	twig
WOER18-L9-SC_65cm	ETH-105424	59.4	47.6	709	22	679	571	leaf
WOER18-L9-E_17cm	ETH-115457	116.4	104.6	1945	23	1942	1750	leaf
WOER18-L9-C_47cm	ETH-115458	294.9	283	4248	25	4863	4657	leaf fragments
WOER18-L9-C_94cm	ETH-115459	342.5	330.7	6153	26	7159	6961	leaf fragments
WOER18-L9-B_4.5cm	ETH-115460	400.2	388.4	8506	29	9540	9477	leaf fragments, needle
WOER18-L9-B_64.5cm	ETH-115461	460.5	419.4	10186	32	11946	11742	leaf fragments, needle

Supplementary Table 3: Mean ages, thicknesses and intensities of events

						turbidite thickness (cm)																	
Event	Mean Age	Min Age	Max Age	Return Time	Unit	L7	L2	L8	L5	L4	L3	L9	L10	historical event	confidence	Known intensity	Normalized Cumulative Thickness	Intensity from Cumulative Turbidite Thickness	Percentage of DA showing an imprint	Intensity from DAs	Intensity from Thresholds	mean intensity
E1	-68	-71	-65	66	3	0	1.5	0.2	0	0	0	0	0	Bad Saag landslide (Daxer et al. 2020)	4		1.46	5.6	25	5.5	6	5.7
E2	-2	-57	99	50	3	0	0	0	7.7	0	0	0	0	Pörtschach & Seefels landslides (Daxer et al. 2020)	4		6.63	6.8	12.5	5.5	6.5	6.3
E3	48	-32	167	45	3	0	0	0	0	0	0	0	0	1976 CE earthquake	5	5.25	0	0	0	0	0	0
E4	93	-4	213	134	3	0	0.7	6.2	0	0	0	0	0	1857 CE earthquake	0	5.5	5.94	6.7	25	5.5	6	6.1
E5	227	104	350	69	3	0	0	0.5	0	0	0	0	0		3		0.43	4.6	12.5	4.8	6	5.1
E6	296	173	418	20	3	0.6	1.5	0	0	0	0	2.2	0	1690 CE earthquake	0	7.75	3.7	6.3	37.5	6.2	8	6.8
E7	316	192	428	80	3	0	0	0	0	0	0	0.5	0		3		0.43	4.6	12.5	4.8	7	5.5
E8	366	239	465	77	3	0	0	0	0	0	0	0.3	0		3		0.26	4.2	12.5	4.8	7	5.3
E9	396	272	489	47	3	0	1.2	0	0	0	0	0	0		3		1.03	5.5	12.5	5.5	6	5.7
E10	443	343	526	70	3	0	0	0	0	0	0	0.4	0		3		0.34	4.4	12.5	4.8	7	5.4
E11	513	423	597	66	3	0	0	1.2	0.5	0	0	1.8	0	1511 CE earthquake	0	7.25	3.01	6.2	37.5	6.2	7	6.5
E12	579	518	660	182	3	0	0	0	0.2	0	0	0	0		3		0.17	3.9	12.5	4.8	6.5	5.1
E13	761	581	965	42	3	33.3	19.9	16.9	30.2	2.3	3.7	7.9	2	1348 CE earthquake	0	8.5	100	9.1	100	9.8	8.5	9.1
E14	803	633	991	13	3	0	0.2	0	0	0	0	0	0		3		0.17	3.9	12.5	4.8	6	4.9
E15	816	643	999	39	3	0	0.4	0.2	0.1	0	0	0	0		2		0.6	4.9	37.5	6.2	6.5	5.9
E16	855	663	1039	79	3	0	1.9	2.6	0.1	0	0	0	0		2		3.96	6.5	37.5	6.2	6.5	6.4
E17	934	736	1116	44	3	0	2.5	1.4	0	0	0	0	0		2		3.36	6.3	25	5.5	6	5.9
E18	978	779	1148	39	3	0	0.2	0.2	0	0	0	0	0		2		0.34	4.4	25	5.5	6	5.3
E19	1017	833	1181	34	3	0	0	0.2	0	0	0	0	0		3		0.17	3.9	12.5	4.8	6	4.9
E20	1051	898	1195	51	3	0	0.2	0.2	0	0	0	0	0		2		0.34	4.4	25	5.5	6	5.3
E21	1102	961	1221	40	3	0	0.2	0.2	0	0	0	0	0		2		0.34	4.4	25	5.5	6	5.3
E22	1142	998	1251	18	3	0	0.5	0.4	0.2	0	0	0	0		2		0.95	5.3	37.5	6.2	6.5	6
E23	1160	1010	1261	81	3	0	2.4	2.5	0.1	0	0	0.1	0		1		4.39	6.5	50	7	7	6.8
E24	1241	1093	1369	6	3	0	1.1	0.8	0	0	0	0	0		2		1.64	5.7	25	5.5	6	5.7
E25	1247	1097	1383	10	3	0	0	0.5	0	0	0	0	0		3		0.43	4.6	12.5	4.8	6	5.1
E26	1257	1105	1407	15	3	0	0.4	1.6	0.1	0	0	0	0		2		1.81	5.8	37.5	6.2	6.5	6.2
E27	1272	1120	1428	8	3	1.2	2.2	1.5	0.1	0	0	0	0		2		4.3	6.5	50	7	8	7.2
E28	1280	1128	1439	15	3	0	0.6	1.1	0	0	0	0	0		2		1.46	5.6	25	5.5	6	5.7
E29	1295	1139	1456	8	3	0	0.2	0.2	0	0	0	0	0		2		0.34	4.4	25	5.5	6	5.3
E30	1303	1146	1467	17	3	0	0	0.2	0	0	0	0	0		3		0.17	3.9	12.5	4.8	6	4.9
E31	1320	1159	1496	24	3	0	0	0	0.1	0	0	0	0		3		0.09	3.3	12.5	4.8	6.5	4.9
E32	1344	1186	1510	99	3	0	0.5	1.6	0.1	0	0	0	0		2		1.89	5.8	37.5	6.2	6.5	6.2
E33	1443	1274	1619	73	3	0	0.3	0.9	0.2	0	0	0	0		2		1.2	5.5	37.5	6.2	6.5	6.1
E34	1516	1337	1687	29	3	0.4	1.6	2.5	0.2	0	0	0	0		2		4.13	6.5	50	7.7	8	7.4
E35	1545	1357	1723	148	3	0	0.6	0	0.1	0	0	0	0		2		0.6	4.9	25	5.5	6.5	5.6
E36	1693	1531	1832	53	3	0	0.5	0.7	0	0	0	0	0		2		1.03	5.3	25	5.5	6	5.6
E37	1746	1606	1872	37	3	0	0.8	0.2	0	0	0	0	0		2		0.86	5.2	25	5.5	6	5.6
E38	1783	1650	1905	103	3	0	1.2	1.1	0	0	0	0	0		2		1.98	5.9	25	5.5	6	5.8
E39	1886	1776	1986	28	3	0	1.6	1.1	0	0	0	0.1	0		1		2.32	6	37.5	5.5	7	6.2
E40	1914	1799	2017	91	3	0	0.8	0.9	0	0	0	0	0		2		1.46	5.6	25	5.5	6	5.7
E41	2005	1870	2162	55	3	0	0.3	0	0	0	0	0	0		3		0.26	4.2	12.5	4.8	6	5
E42	2060	1910	2234	24	3	0	0.3	0	0	0	0	0	0		3		0.26	4.2	12.5	4.8	6	5
E43	2084	1927	2258	33	3	0	0.5	0	0	0	0	0	0		3		0.43	4.6	12.5	4.8	6	5.1
E44	2117	1949	2307	86	3	0	1.5	0	0	0	0	0	0		3		1.29	5.5	12.5	4.8	6	5.4
E45	2203	2022	2393	32	3	0	0.7	0	0	0	0	0	0		3		0.6	4.9	12.5	4.8	6	5.2
E46	2235	2055	2425	64	3	0	0.4	0	0	0	0	0	0		3		0.34	4.4	12.5	4.8	6	5.1
E47	2299	2113	2487	86	3	0.4	0.6	0	0	0	0	0	0		2		0.86	5.2	25	5.5	8	6.2
E48	2385	2198	2567	171	3	0	0.5	0	0	0	0	0	0		3		0.43	4.6	12.5	4.8	6	5.1
E49	2556	2365	2700	86	3	0	0.7	0.6	0	0	0	0	0		2		1.12	5.4	25	5.5	6	5.6
E50	2642	2488	2735	116	3	0	0.8	1.1	0	0	0	0	0		2		1.64	5.7	25	5.5	6	5.7
E51	2758	2595	2883	70	3	0	0.3	0.2	0.4	0	0	0	0		2		0.77	5.1	37.5	6.2	6.5	5.9

E52	2828	2654	2962	98	3	0	0.3	0.6	0.2	0	0	0	0		2		0.95	5.3	37.5	6.2	6.5	6
E53	2926	2776	3055	404	3	0	0.2	0	0	0	0	0	0		3		0.17	3.9	12.5	4.8	6	4.9
E54	3330	3226	3434	43	2	0.1	0.1	0	0	0	0	0	0		2		0.17	3.9	25	5.5	8	5.8
E55	3373	3284	3474	54	2	0.2	0.4	0	0.7	0	0	0.1	0		1		1.2	5.5	50	7	8	6.8
E56	3427	3367	3539	30	2	0	0	0	0.2	0	0	0	0		3		0.17	3.9	12.5	4.8	6.5	5.1
E57	3457	3388	3572	12	2	0	0	0	0.3	0	0	0	0		3		0.26	4.2	12.5	4.8	6.5	5.2
E58	3469	3393	3590	2	2	0	0	0	0.1	0	0	0	0		3		0.09	3.3	12.5	4.8	6.5	4.9
E59	3471	3393	3594	8	2	0	0.4	0.4	1.4	0	0	0	0		2		1.89	5.8	37.5	6.2	6.5	6.2
E60	3479	3396	3610	30	2	2.5	1.2	1.9	6.9	0	0	0.2	0		1		10.93	7.3	62.5	7.7	8	7.7
E61	3509	3413	3658	39	2	0.3	0.2	0	0	0	0	0	0		2		0.43	4.6	25	5.5	8	6
E62	3548	3432	3714	19	2	1.5	3.7	2.1	6.6	0	0	0.6	0.8		1		13.17	7.5	75	8.4	8.5	8.1
E63	3567	3447	3741	30	2	0	0.3	0	0	0	0	0	0		3		0.26	4.2	12.5	4.8	6	5
E64	3597	3466	3782	10	2	2.7	0.9	0	1.5	0	0	0	0		2		4.39	6.5	37.5	6.2	8	6.9
E65	3607	3472	3801	428	2	0.7	0.5	0	0	0	0	0	0		2		1.03	5.3	25	5.5	8	6.3
E66	4035	3810	4268	599	2	0	0.4	1.6	0	0	0	0	0		2		1.72	5.8	25	5.5	6	5.8
E67	4634	4523	4767	2138	2	0.1	0.3	0.9	0	0	0	0.5	0.4		1		1.89	5.8	62.5	7.7	8.5	7.3
E68	6772	6504	6990	515	2	0.2	2.8	1.1	0	0	0	0.5	0		1		3.96	6.5	50	7	8	7.2
E69	7287	7077	7537	51	2	0	0	0.6	0	0	0	0	0		3		0.52	4.8	12.5	4.8	6	5.2
E70	7338	7113	7597	46	2	0	0	0.1	0	0	0	0	0		3		0.09	3.3	12.5	4.8	6	4.7
E71	7384	7148	7649	18	2	0	0	0.2	0	0	0	0	0		3		0.17	3.9	12.5	4.8	6	4.9
E72	7402	7158	7675	272	2	0	0	0.2	0	0	0	0	0		3		0.17	3.9	12.5	4.8	6	4.9
E73	7674	7410	7951	30	2	0	0	0.2	0	0	0	0	0		3		0.17	3.9	12.5	4.8	6	4.9
E74	7704	7430	7989	33	2	0	0	0.2	0	0	0	0	0		3		0.17	3.9	12.5	4.8	6	4.9
E75	7737	7468	8008	230	2	0	0	0.2	0	0	0	0	0		3		0.17	3.9	12.5	4.8	6	4.9
E76	7967	7711	8188	2466	2	0	1.3	1.2	0	0	0	0	0		2		2.15	6	25	5.5	6	5.8
E77	10433	9950	10966	715	2	0.3	2	5.7	0.2	0	0	0	0		2		7.06	6.9	50	7	8	7.3
E78	11148	10771	11500	350	2	0	0	0	0.2	0	0	0.2	0		1		0.34	4.4	25	5.5	7	5.6
E79	11498	11343	11716	90	2	10.5	39.1	18.2	32.3	0	1.4	29.2	8.1		1		119.45	9.3	87.5	9.1	8.5	9
E80	11588	11398	11767	1056	2	0	0	0	0	0	0	0	0.7		3		0.6	4.9		0	8.5	4.5
E81	12644	12167	13105	145	2	0.3	0.5	2.1	0.7	0	0	0.3	0		1		3.36	6.3	62.5	7.7	8	7.3
E82	12789	12253	13276	105	2	0.3	0.1	0.3	0.1	0	0	0	0		2		0.6	4.9	50	7	8	6.6
E83	12894	12385	13325	49	2	0.1	0.5	0	0	0	0	0	0		2		0.52	4.8	25	5.5	8	6.1
E84	12943	12430	13358	56	2	0	0.4	0	0	0	0	0	0		3		0.34	4.4	12.5	4.8	6	5.1
E85	12999	12472	13413	42	2	0.3	0.8	0	0	0	0	0	0		2		0.95	5.3	25	5.5	8	6.3
E86	13041	12514	13460	35	2	0.8	0.8	0	0	0	0	0	0		2		1.38	5.6	25	5.5	8	6.4
E87	13076	12559	13467	201	2	0	0.2	0	0	0	0	0	0		3		0.17	3.9	12.5	4.8	6	4.9
E88	13277	12790	13587	437	2	0	0.7	1	0	0	0	0	0		2		1.46	5.6	25	5.5	6	5.7
E89	13714	13534	13965	428	2	0	0.8	0.9	0	0	0.8	0.3	0		1		2.32	6	50	7	8.5	7.2
E90	14142	13803	14583		1	221.6	87.7	204.6	374.6	272.2	41.2	196.2	289.1		1		1451.98	11.3	100	9.8	8.5	9.9

Supplementary Table 4: Statistical analysis

Subset	min years	max years	Time span (years)	Nr. intervals	Median (yr)	Mean (yr)	Standard deviation	CoV	Burstiness	Skewness	C&O value	C&O p-value	Reject Poissonian via exponential distribution? C&O test (95%)	Lcorr KS p-value	Reject Poissonian via exponential distribution? LCorr KS test (95%)	Best Fitting Distribution	p-value of best fit	2nd Best Fitting Distribution	p-value of 2nd best fit	Conclusion regarding recurrence pattern
all, intensity >= V	93	14142	14049	43	103	327	530	1.6228	0.237456154	2.8291	-32.78	2.20E-16	yes	4.00E-04	yes	Lognormal	0.90389	Weibull	0.66682	bursty
all, intensity >= VI	296	14142	13846	22	246	629	866	1.3757	0.158142863	2.4203	-13.644	0.0225	yes	0.0602	yes	Gamma	0.96711	Lognormal	0.9579	bursty
all, intensity >= VII	296	14142	13846	10	1068	1385	1729	1.2489	0.110676331	2.2045	-2.0553	0.6105	no	0.3178	no	Gamma	0.79893	Lognormal	0.60815	bursty/ Poissonian?
unit II & III, intensity >= V	93	13714	13621	42	101	324	536	1.654	0.246420497	2.8162	-33.955	2.20E-16	yes	4.00E-04	yes	Lognormal	0.89429	Weibull	0.63794	bursty
unit II & III, intensity >= V1/2				41	103	332	541	1.6278	0.238907071	2.779	-31.759	5.00E-04	yes	4.00E-04	yes	Lognormal	0.82891	Weibull	0.6739	bursty
unit II & III, intensity >= VI	296	13714	13418	21	244	639	886	1.3866	0.161987765	2.3477	-14.293	0.011	yes	0.028	yes	Weibull	0.91031	Gamma	0.90971	bursty
unit II & III, intensity >= VI1/2				13	1037	1032	1044	1.0116	0.005766554	1.4561	-0.79709	0.877	no	0.5936	no	Exponential	0.7756	Gamma	0.76714	Poissonian
unit II & III, intensity >= VII	296	13714	13418	9	1070	1491	1799	1.2068	0.09371035	2.0609	-1.8288	0.6585	no	0.3544	no	Gamma	0.66457	Exponential	0.5901	bursty/ Poissonian?
unit II, intensity >= V	3373	13714	10341	17	236	608	755	1.2405	0.107342111	0.26622	-11.213	0.024	yes	0.1792	no	Gamma	0.95161	Weibull	0.93495	bursty
unit II, intensity >= V1/2	3373	13714	10341	17	236	608	755	1.2405	0.107342111	0.26622	-11.213	0.024	yes	0.1792	no	Gamma	0.95161	Weibull	0.93495	bursty
unit II, intensity >= VI	3471	13714	10243	13	210	788	1078	1.3675	0.155227033	1.8627	-13.499	0.003	yes	0.0262	yes	Gamma	0.79456	Weibull	0.777	bursty
unit II, intensity >= VI1/2				8	1068	1279	1168	0.91318	-0.045379943	1.2507	-0.31294	0.948	no	0.1458	no	Gamma	0.44875	Exponential	0.36882	Poissonian
unit II, intensity >= VII	3479	13714	10235	6	1078	1706	2047	1.1998	0.090826439	2.2133	-0.65951	0.853	no	0.1668	no	Exponential	0.38632	Gamma	0.35417	Poissonian
unit III, intensity >= V	93	2828	2735	24	88	114	94	0.82923	-0.093356221	1.5072	7.0165	0.262	no	0.1684	no	Gamma	0.87276	Weibull	0.54712	weakly periodic
unit III, intensity >= V1/2				23	90	119	95	0.80266	-0.109471559	1.1522	7.211	0.24	no	0.0968	no	Gamma	0.85367	Weibull	0.44061	weakly periodic
unit III, intensity >= VI	296	2299	2003	7	244	286	231	0.8102	-0.104850293	2.0567	3.8201	0.2465	no	0.322	no	Lognormal	0.93518	Gamma	0.82201	weakly periodic
high frequency period II	3373	3607	234	5	49	47	39	0.8261	-0.095230272	1.2524	1.3271	0.6715	no	0.7076	no	Exponential	0.84764	Weibull	0.77495	
